# Exploring mudbrick architecture and its re-use in Artaxata, Armenia, during the 1st millennium BC. A multidisciplinary study of earthen architecture in the Armenian Highlands

**DOI:** 10.1371/journal.pone.0292361

**Published:** 2023-10-13

**Authors:** Marta Lorenzon, Benjamín Cutillas-Victoria, Elisabeth Holmqvist, Myrsini Gkouma, Luc Vrydaghs, Achim Lichtenberger, Torben Schreiber, Mkrtich Zardaryan

**Affiliations:** 1 Department of Cultures, University of Helsinki, Helsinki, Finland; 2 NCSR Demokritos, Athens, Greece; 3 Murcia University, Murcia, Spain; 4 M.H. Wiener Laboratory for Archaeological Science, American School of Classical Studies at Athens, Athens, Greece; 5 AMGC—Vrije Universiteit Brussel, Brussels, Belgium; 6 University of Münster, Münster, Germany; 7 National Academy of Sciences of the Republic of Armenia, Yerevan, Armenia; Universita degli Studi di Milano, ITALY

## Abstract

Mudbrick constructions are extremely common in ancient western Asia, including the 1st millennium structures of the southern Caucasus and Armenian highlands. However, in the Caucasus the geoarchaeological study of these materials to provide insight into building practices and social structure is a topic little researched, especially when focusing on the *longue durée*. Artashat/Artaxata (Ararat region, Armenia) was the capital of the Armenian Kingdom of the Artaxiads, founded in the eighties of the 2nd century BC, but even before this the site was occupied in the Chalcolithic period, (ca. 5200–3500 BC), Early Iron Age (ca. 1200–900 BC) and in the Urartian period (ca. 800–600 BC) as well. All the previous occupation phases showed communities that made extensive use of earthen constructions as determined during past and recent archaeological excavations. This multidisciplinary study seeks to examine mudbrick architecture as a proxy for environmental and social interactions during the 1st millennium BC combining geoarchaeology, archaeobotany and building archaeology. We analyzed changes and continuities in architectural form and practices, alongside reconstruction of technological and social processes, to identify issues of raw material procurement, attestation of re-use, and consistency of building practices. The results of the geoarchaeological analysis of the earthen building materials used in different parts of the ancient city point to a re-use of materials over time.

## Introduction

Compared to Mesopotamia and the Levant, mudbrick constructions in the Armenian highlands and Caucasus have been the focus of limited research, mostly at the macroscopic and chronological level [1–3]. The importance of architecture, especially earthen architecture, in shedding light on past social practices and interactions is well attested [4–6], both as a proxy to understand past environmental changes and to determine past social practices [7, 8]. Nevertheless, Classical period buildings are often not given the same attention as prehistoric ones in the analysis of earthen building materials and their labor organization.

**Fig 1:**
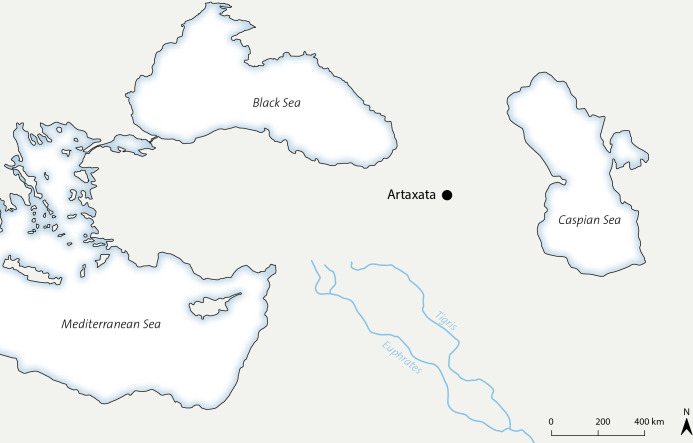
Map representing the site location and morphology (credit: Maija Holappa; source: USGS EROS, public domain).

The Armenian-German Artaxata Project (AGAP) launched in 2018 at Artaxata/Artashat (Ararat region, Armenia) focuses on the urbanism of the capital of the Armenian Kingdom of the Artaxiads, founded in the eighties of the 2nd century BC. In Armenia, specifically Artaxata, the Classical period ranges from the 2nd century BC to the 4th century AD. This project builds on the results of the previous Armenian excavations of the 1970s [9–11], which have studied the urban development of the numerous hills that constitute the ancient city (Fig 1).

Our research first aims at bringing forward the analysis of earthen building materials at the site of Artaxata, located in the Ararat Valley of central Armenia. Second, we compare construction practices between the two main phases of site occupation during the 1st millennium BC: Urartian and Hellenistic. This comparison allows us to investigate differences and similarities in sources of raw material procurement and building practices, but especially to provide a new methodology to assess the issue of re-use of mudbricks from archaeological context. This is a particularly important question, as currently there is no such methodological framework for the determination of earthen building material (henceforth EBM) re-use.

The re-use of EBM, specifically mudbrick, is an essential and understudied topic in archaeological research. Their re-employment in buildings over the centuries does not follow the same pattern of other *spolia* and ceramic building materials such as *laterizio*. Re-used mudbricks are taken from previous buildings, broken down and remixed with additional water and temper to mold new bricks. In contrast to fired bricks in which re-use is easily detectable, only rarely are mudbricks taken from a building to be directly employed in another construction (author personal observation during her work in Egypt). More commonly they are worked and reshaped thus fully recycling the materials, but also re-initiating the manufacturing process [12]. This type of intensive recycling can still be considered re-use with the caveat that it is incredibly challenging to trace and identify in the archaeological record.

The traditional mudbrick *chaîne opératoire* consists of collection of raw materials, thus soil, water, and human-induced inclusions used as degreasers such as the more commonly used vegetal temper, but also shell fragments, sand, and crushed plaster. The mixing, molding, and drying activities have been extensively described [13, 14], indicating how the analysis of the *chaîne opératoire* can provide useful information regarding the procurement of raw materials within a community [8, 15], the presence of skilled or unskilled workforces [6, 16, 17], and the development of apprenticeship within a community [14]. The follow up study of how earthen structures decay, collapse and participate in postdepositional processes brings forth further knowledge regarding the cycle of architecture and materials collapse [18, 19]. Here, we argue that an integrated approach that combines geoarchaeology, micromorphology and archaeobotany provides us with the necessary parameters to recognize re-used building materials.

One innovative aspect of the present study is the phytolith analysis of EBM thin sections. While the observation of opal of biological origin within petrographic thin sections is not uncommon [20, 21], the systematic examination of phytoliths in ceramic thin sections is restricted to a few case studies [see for instance 22–27]. Even more infrequent are studies elaborating on the phytolith content of construction remains such as bricks and cement [18, 28–33]. Most of these studies extract phytoliths which ends in the loss of part of the phytolith’s context.

Phytolith taphonomy is a complex matter [for a discussion of this issue see 34–36]. Prior to any taphonomic process such as erosion, corrosion or transport, decomposition of organic matter needs to occur to release phytoliths. Decomposition of the organic matter can occur either before or after the incorporation of the plant fragment into an archaeological record. When decay of the plant fragment takes place after its incorporation into the archaeological object, the relative distribution of the phytoliths, as observed while they are in anatomical position, should be preserved, provided that no post-depositional perturbation occurred. This relative distribution, together with its context, is destroyed when phytoliths are removed for analysis using typical extraction processes. On the contrary, they are preserved in thin sections.

Phytolith analysis on thin sections is a technique that inventories the distribution patterns of the phytoliths within archaeological contexts as well as the phytoliths composing each of these patterns. Each pattern (namely, Isolated, Clustered and Articulated (for a definition see [37, 38]) hides a different history. The inventory of each distribution pattern together with the phytolith composing each of them, enables to discriminate phytoliths sharing (or not) a common (post)depositional history and botanical origin [37, 39, 40]. Concerning ceramic thin sections, it discriminates phytoliths observed in voids from those in the clay matrix. Previous taphonomic research determined phytoliths within voids derive from plant material selected as temper while those in the matrix relate to the natural composition of the clay [37]. Such an approach successfully contributed to the identification of ware source areas and the reconstruction of the *chaîne opératoire* [41].

### Archaeological background

The Hellenistic city Artaxata was founded in the 180s BC by the Armenian king Artaxias I as the capital of his kingdom at a location which was said to be previously uninhabited [42, 43]. However, recent archaeological research has shown that the city was the site of a considerable Urartian settlement and Artaxias I resettled the apparently abandoned site [44].

The Hellenistic city was located on 17 natural limestone hills which rise from the Ararat Valley and its lower city stretched into the plain. The river Araxes passes the west and south of the city. The rivers Metsamor and Azat, which flow into the Araxes, come from the northeast, while the Vedi waters the south of the city. The Ararat Valley is a rich and fertile alluvial plain with clay deposits used for pottery and mudbrick production [45]. The capital city of Artaxata soon became a major metropolis in Armenia and a melting pot of a variety of interactions with the Mediterranean, the Levant, Iran, Mesopotamia and the northern Caucasus. Architecturally, Artaxata was strongly reliant on local construction techniques and most of the buildings were made of mudbricks placed on low stone foundations. In the archaeological record, at least the lower courses of the mudbrick walls can be found intact, even if most of the former walls are eroded and deformed.

**Fig 2:**
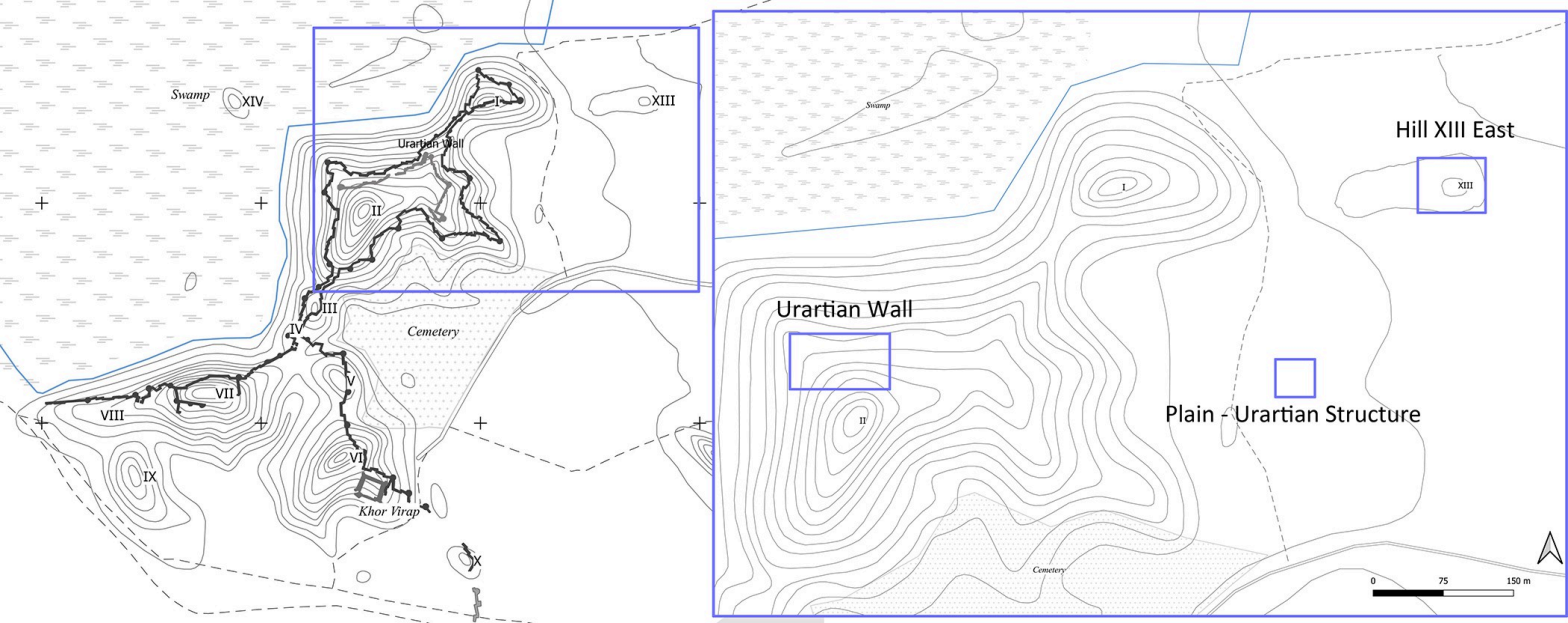
Map of excavation areas on the hills of Artaxata showing (A) the full extension of Artaxata site with its hills and (B) zoom in in the area investigated in this article, specifically Hill II, Hill XIII and the plain (Credit: Torben Schreiber).

Armenia was affected heavily by the antagonism between Rome and Parthia but especially under Tigran II was a mighty transregional power. The city experienced several violent destructions, among them around 66 BC a partial destruction during Tigran the Younger’s rising and in AD 59 by Corbulo. These destructions seem to be attested in the archaeological record. In this study mudbrick samples are included which stem from the Urartian period and from the first two main phases of the Hellenistic settlement, from Phase I (180s to approx. 66 BC) and Phase II/III (approx. 66 BC to AD 59). These mains phases were identified by stratigraphic excavations and a 14C sampling program. The data was mainly assembled on Hill XIII which is the main site of the Armenian-German Artaxata Project (AGAP) (Fig 2).

Although our research focuses on the Hellenistic Lower City and its three-phase development (Phases I, II and III) during the Hellenistic period, Artaxata’s nature as a multi-period site is pertinent to our study. Besides the dense Hellenistic settlement, traces from the Chalcolithic, Middle Bronze Age, and–as previously mentioned–Urartian period have been documented [46]. During this work, remains of an apparently extensive Urartian settlement were found on Hill XIII and in the southern plain, alongside the previously documented citadel on Hill II [9–11].

More importantly the radiocarbon dates provide us with solid evidence that these two distinct periods of occupation, Urartian and Hellenistic, occurred in the same area of the settlements indicating for instance that Trench 11 areas present Hellenistic occupation built directly on top of Urartian remains, even if the exact stratigraphic sequence needs to be further investigated (Table 1. Data in S1 File).

**Table 1:** Set of 14C dates calibrated with the OxCal v4.4 software (IntCal20 Northern Hemisphere).

Lab No	Feature No.	14C Age [yr BP]	±	δ13C AMS [‰]	Probability 68%	Probability 95%	C [%]	Material	Stratigraphical relation to samples	Chronology
48747	ART20T309	2117	25	-24.5	cal BC 171–58	cal BC 337–51	54.7	charcoal	Above AA4	Hellenistic Period (Phase I or shortly before)
48751	ART20T320	2164	23	-22.6	cal BC 347–168	cal BC 353–107	72.3	charcoal	Above AA4	Hellenistic Period (Phase I or shortly before)
48748	ART20T323	2148	25	-23.2	cal BC 344–120	cal BC 350–56	63.5	charcoal	Underneath AA4	Hellenistic Period (Phase I or shortly before)
48753	ART20T1107	2148	23	-21.0	cal BC 343–123	cal BC 350–56	55.6	charcoal	Same as AA5 and AA6	Hellenistic Period (Phase I collapse)
48754	ART20T1107	2095	24	-26.2	cal BC 150–53	cal BC 192–5	60.5	charcoal	Same as AA5 and AA6	Hellenistic Period (Phase I collapse)
48755	ART20T1107	2134	22	-16.0	cal BC 335–108	cal BC 344–55	25.7	Bone	Same as AA5 and AA6	Hellenistic Period (Phase I collapse)
53468	ART21T1115	2576	21	-23.2	cal BC 792–776	cal BC 804–675	2.1	charcoal	Same complex as AA11-AA14	Urartian Period
53469	ART21T1126	2616	19	-4.4	cal BC 805–793	cal BC 808–780	19.5	charcoal	Same complex as AA11-AA14	Urartian Period

#### Geological background

**Fig 3:**
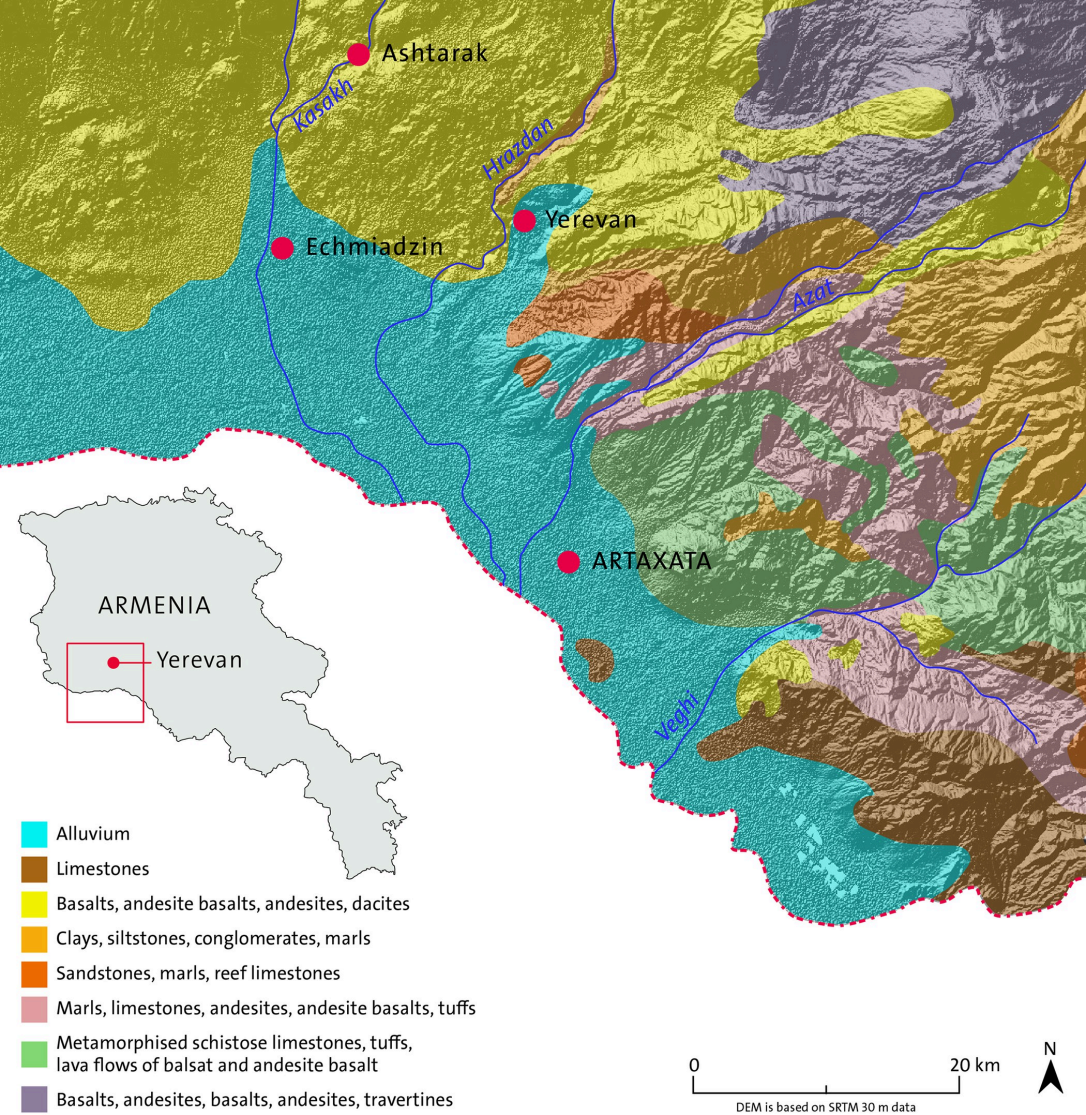
Geological map of Armenia (drawing: Maija Holappa; adapted after https://doi.org/10.3133/ofr98479; public domain).

Armenian geological map is complex and multifaceted. Most of the area around Artaxata is characterized by diatomite clays and Quaternary lacustrine alluvium (Fig 3). Paleogene and sandstone deposits alternating marls, sandstones, siltstones, tuffs and gravel surrounded the site to the North and the North- East. Directly to the East of the site, the geological landscape includes cretaceous deposits of metamorphic schistose limestones, tuff conglomerates and basalts, while in the south we have cretaceous carbonate and limestones. Ting et al. conducted a sampling of the area to study the raw materials for ceramic production in Artaxata, which is the baseline for the geological analysis in this article [45].

### Excavation area

The main focus of the AGAP, starting in 2018, has been on Hill XIII and the adjacent plain to the south. The hill is located at the transition from the Upper to the Lower City of Hellenistic Artaxata. The starting point for the excavation work is the results of geophysical prospection, which revealed several building structures in the Lower City [47]. In five excavation campaigns, Hill XIII, an anomaly in the form of a dotted line to the north of it, and a hall-like structure recognizable in the magnetics in the southern plain were investigated.

While the anomaly to the north turned out to be the remains of pillars of an unfinished Roman aqueduct bridge [48], the remains of large-scale constructions were found on Hill XIII. The most significant features, which were to a certain extent previously evident in the magnetics, are quarry stone wall bases, some of which overlap, revealing a relative chronological sequence. Almost the entire eastern summit of the saddle-shaped Hill XIII and its slopes as well as the depression on the western hilltop are covered with a massive layer of collapsed mudbrick, deriving from the walls that were built on the quarry stone bases [49].

Construction here took place in two main phases, which could be firmly dated using 14C samples. Phase I begins around 180 BC with the construction of a presumably sacred building with a broad central room, furnished with rich stucco decoration [48]. Traces of fire and partial destruction were recognized, which occurred around the middle of the 1st century BC, not impacting the mudbrick walls. In the sub-phase of Phase I (Ib), a change in function to domestic architecture becomes apparent. This change in function becomes clear in Phase II (from the middle of the 1st century BC). So far, two *corridor houses* separated by an alley have been excavated from this phase. Phase II was followed by the almost congruent Phase III. Structural and functional changes can hardly be observed between the two phases. The end of Phase III is marked by the destruction under Corbulo in 59 AD. The small number of finds allows the assumption that the inhabitants had already left their dwellings before the destruction. Hill XIII was then never resettled but visited only sporadically and the dwellings left behind weathered away.

Already in 2018, remains of past mudbrick architecture were documented in a test trench in the area adjoining the eastern hilltop to the north [50]. When work was resumed in 2022 it turned out that we are dealing with a massive filling underneath a retaining wall of Phase I. According to the pottery finds the material used for leveling this area must come from the Urartian period.

Already in 2018, a structure with a length of over 60 m was discovered on the magnetogram in the plain in the southern part of the investigation area [44]. This was partially excavated in 2021. In an area of 195 m^2^ the northwest corner of the structure and a three-part double gate in the center were uncovered. The function of this structure is still unclear, but on the basis of the 14C data, it can be dated with certainty to the Urartian period around 800 BC. This represents an important finding in pre-Hellenistic urban history and the first monumental complex from that period in the study area.

In addition to the abovementioned features and some scattered finds, a massive mudbrick wall on the northern slope of Hill II is a particular testimony to the Urartian past of the site. It is assumed that the remains still visible today were part of an Urartian fortress with a size of 2.5 to 2.6 ha [51] (Fig 2).

## Materials and methods

**Fig 4:**
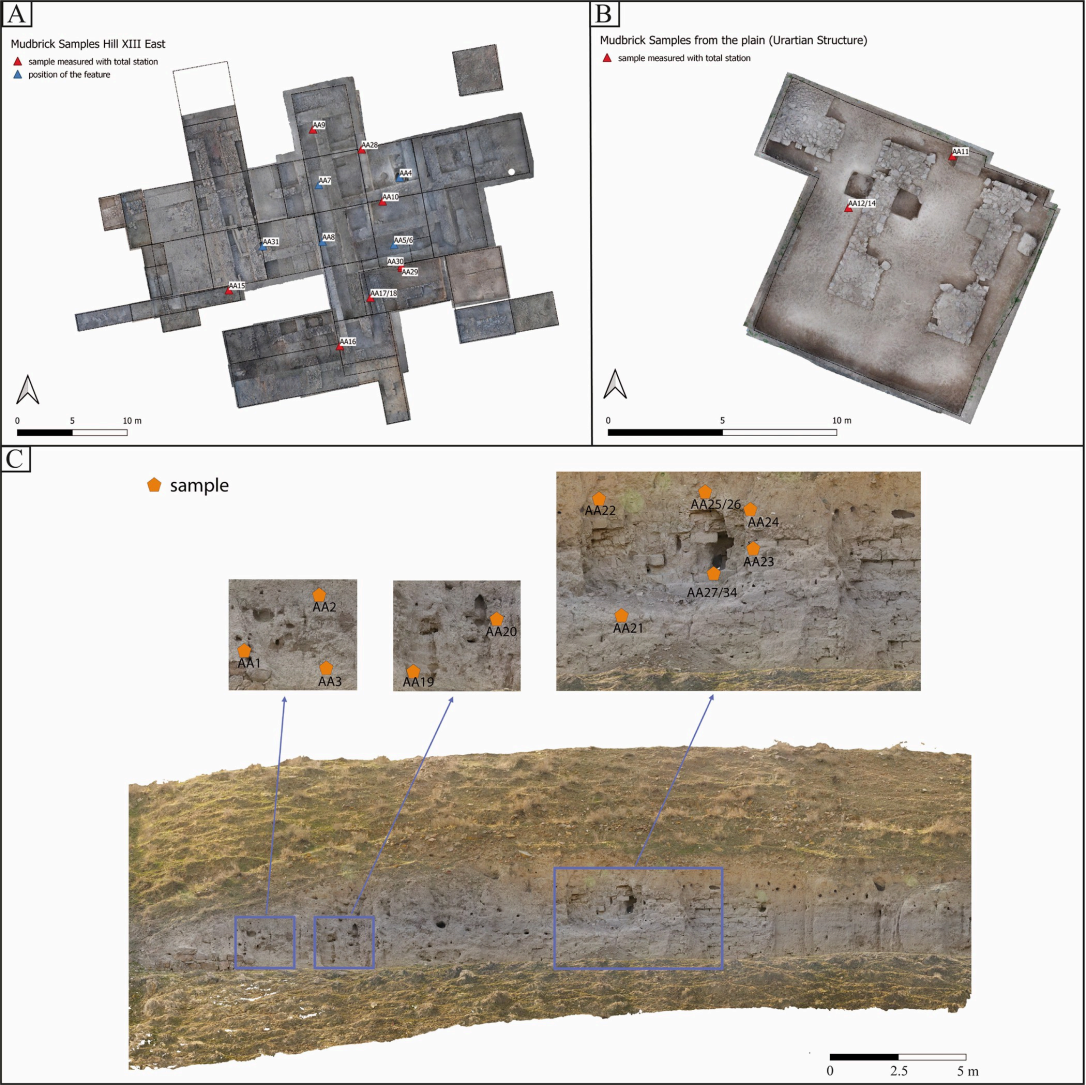
Sampling areas and location within the trenches in Artaxata, Armenia. A. Samples’ location in Hill XIII; B. Samples’ location in the plain; C. Samples’ location on the Urartian wall on Hill II (Credit: Torben Schreiber).

Sampling took place from different sectors of Artaxata that have been recently excavated and from the Urartian defensive wall, excavated in the 1970s. When possible, the samples were selected from multiple rows, top to bottom, of the Urartian defensive wall (Hill II), preferably from external walls of the buildings identified on Hill XIII, Trench 11 in the plain, which presented both Urartian and Hellenistic levels, and trench 1–4, 6, 8, which displayed Hellenistic occupation levels (Fig 4). Due to the continuous rebuilding and levelling actions during the Hellenistic period, often only limited mudbrick remains such as one row of mudbrick, were preserved in situ for sampling. Therefore, the 34 samples include the multiple phases of site occupation, specifically Urartian and Hellenistic, including phase I, II and III registered on Hill XIII (Table 2).

**Table 2:** Artaxata mudbrick and soil samples.

Sample ID	Trench	Period	Sector	description	Period	Feature No.
AA1	Urartian wall	Urartian	Hill II	Mudbrick	Urartu	-
AA2	Urartian wall	Urartian	Hill II	Mudbrick	Urartu	-
AA3	Urartian wall	Urartian	Hill II	Mudbrick	Urartu	-
AA4	trench 3	Hellenistic	Hill XIII	Mudbrick	Phase I or prior (underneath floor of Phase I)	ART20T0322
AA5	trench 11	Hellenistic	Hill XIII	Mudbrick	Phase I (collapse/destruction)	ART20T1107
AA6	trench 11	Hellenistic	Hill XIII	Mudbrick	Phase I (collapse/destruction)	ART20T1107
AA7	trench 2	Hellenistic	Hill XIII	Mudbrick	collapse phase II or phase III	ART20T0210
AA8	trench 1	Hellenistic	Hill XIII	Mudbrick	collapse phase II or phase III	ART20T0109
AA9	trench 4	Hellenistic	Hill XIII	Mudbrick	Mudbrick from phase II wall	ART20T0403
AA10	trench 3	Hellenistic	Hill XIII	Mudbrick	Mudbrick from phase II wall	ART20T0301
AA11	trench 11	Urartian	plain	Mudbrick	Urartu Mudbrick from collapse of wall of Urartian structure	ART21T1133
AA12	trench 11	Urartian	plain	Mudbrick	Urartu Mudbrick from collapse of wall of Urartian structure	ART21T1110
AA13	trench 11	Urartian	plain	Mudbrick	Urartu	
AA14	trench 11	Urartian	plain	Mudbrick	Urartu Mudbrick from collapse of wall of Urartian structure	ART21T1110
AA15	trench 4	Hellenistic	Hill XIII	Mudbrick	Intermediate clay layer between wall of Phase II and Phase III	ART21T0406
AA16	Trench 9(2020)	Hellenistic	Hill XIII	Mudbrick	Intermediate clay layer between wall of Phase II and Phase III	ART20T0903
AA17	trench 8	Hellenistic	Hill XIII	Mudbrick	Melted mudbrick, collapse, filling, assigned to Phase II or Phase III	ART21T0815
AA18	trench 8	Hellenistic	Hill XIII	Mudbrick	Melted mudbrick, collapse, filling, assigned to Phase II or Phase III	ART21T0815
AA19	Urartian wall	Urartian	Hill II	Mudbrick	Urartu	
AA20	Urartian wall	Urartian	Hill II	Mudbrick	Urartu	
AA21	Urartian wall	Urartian	Hill II	Mudbrick	Urartu	
AA22	Urartian wall	Urartian	Hill II	Mudbrick	Urartu	
AA23	Urartian wall	Urartian	Hill II	Mudbrick	Urartu	
AA24	Urartian wall	Urartian	Hill II	Mudbrick	Urartu	
AA25	Urartian wall	Urartian	Hill II	Mudbrick	Urartu	
AA26	Urartian wall	Urartian	Hill II	mud mortar	Urartu	
AA27	Urartian wall	Urartian	Hill II	Mudbrick	Urartu	
AA28	SO1	Hellenistic	Hill XIII	Soil-Mudbrick	Collapse of Phase I wall (?)	
AA29	trench 8	Hellenistic	Hill XIII	Installation/ Kiln lining	Kiln wall; on top of Phases II and III; supposedly medieval	ART21T0812
AA30	trench 8	Hellenistic	Hill XIII	Upper lining kiln	Kiln wall; on top of Phases II and III; supposedly medieval	ART21T0812
AA31	trench 6	Hellenistic	Hill XIII	Fired/burned brick	Destruction layer close to the basin ART19T0609. Brick belonged to basin	ART19T0603
AA32	trench 3	Hellenistic	Hill XIII	Mudbrick	Soft soil underneath the topsoil on both sides of a Phase Ib wall	ART21T0303
AA33	trench 3	Hellenistic	Hill XIII	Mudbrick	Soft soil underneath the topsoil on both sides of a Phase Ib wall	ART21T0303
AA34	Urartian wall	Urartian	Hill II	Mudbrick	Urartu	

The 34 samples underwent geochemical and petrographic analysis. Based on the results and material preservation, we sub-selected 17 samples to perform granulometric analysis, calcimetry, loss on ignition and four for phytolith analysis.

### Methodology

Major, minor, and trace element concentrations of 34 bulk samples were analyzed with a Rigaku NEX-DE VS bench-top ED-XRF spectrometer housed in the University of Helsinki Laboratory of Archaeology. The instrument was operated in point analysis mode, using 1 mm beam diameter and a camera view to select inclusion-free points to acquire paste geochemical compositions. The results are normalized means of 3–5 analyzed points per sample, measured in helium atmosphere, using a tube voltage of 60 kV, 35 kV, 6.5 kV and acquisition times of 60, 60 and 100 s for high-Z, mid-Z and low-Z elements, respectively. The results were quantified by the instrument’s software and fundamental parameters; standard reference materials Burnt Refractory NIST 76a and Brick clay NIST 679 were analyzed with the samples to control data precision and accuracy. The ED-XRF dataset was processed with the IBM SPSS 28 software, the compositional groups are based on cluster analysis (CA) groupings using the Centroid Clustering method run with the elemental concentrations of MgO, Al_2_O_3_, SiO_2_, K_2_O, CaO, TiO_2_, MnO, Fe_2_O_3_, NiO, ZnO, Rb_2_O, SrO, Y_2_O_3_, and ZrO_2_ detected in the entire sample set (P_2_O_5_, SO_3_, and Cl were excluded due to suspected contamination).

Loss on Ignition (LOI) was used to quantify organic matter content as the samples were pre-dried for 24 hrs, cooled, and weighed. Then samples were placed in the furnace at 500°C for at least 6 hrs, cooled and weighed again for organic loss.

CaCO_3_ was calculated through an automated calcimeter, GEO-RS calcimeter, capable of measuring the percentage of carbonate from the pressure increase within a sealed circuit based on the carbon dioxide developed from the reaction of 0.500 g of sample with 5 ml of 10% (v/v) HCl. The calcimeter was calibrated using 0.500 g of 99.95% pure calcium carbonate (produced by Alfa Aesar) and 5 ml of 10% HCl. The calibration was repeated after every 5 analyses performed.

Petrographic and mineralogical analyses of the thin sections were conducted with a Leica DM2000 polarized light microscope with an attached digital camera, working with a magnification between ×5 and ×40. The samples were analyzed and grouped following the system of structure and component descriptions [for detailed methodology see 52–56].

A few samples were selected for micromorphological analysis. They were oven dried at 50°C and impregnated with polyester resin without disturbing their original structure. When they were solidified, they were cut with a rock saw into slabs of 2 cm thickness and with gradual cutting and polishing, thin sections of 30 μm thickness (5×7cm) were created. The initial processing of the samples was conducted at the M.H. Wiener Laboratory of Archaeological Science, ASCSA, while the thin sections were produced at Quality Thin Sections (Tucson, Arizona). In total, four thin sections were produced. The thin sections were photographed in high resolution [see 57]; they were studied under the stereoscope and the polarized microscope in magnifications ranging from ×1.25 to ×40 [55, 58].

In addition, four petrographic thin sections were also used to study phytoliths and testing the viability of the methods for earthen architecture. Observations were conducted on a Zeiss Aksioscope under PPL and XPL [59]. When needed, additional observations were conducted under UV and Blue lights. Thin sections were scanned along two horizontal and vertical lines at magnifications ×100, ×160, ×200, ×500, and ×800. The purpose of the scanning at low magnifications (×100, ×160, ×200) is to detect the presence of phytoliths in voids. Scanning at higher magnifications (×500 and ×800) aims either at naming phytoliths observed in voids or detecting phytoliths in the fired clay. Indeed, due to a potential wedging effect [58], phytoliths can be masked by the fine fraction. Naming of phytoliths follows ICPN 2.0 (ICPT [60, 61]). As recommended for the analysis of soil thin sections [62], attention is also paid to the presence/absence of opal residues of biological origin (diatoms, chrysophyceae cysts, sponge spicules, external amiboid skeletons).

## Results

### ED-XRF results

**Fig 5:**
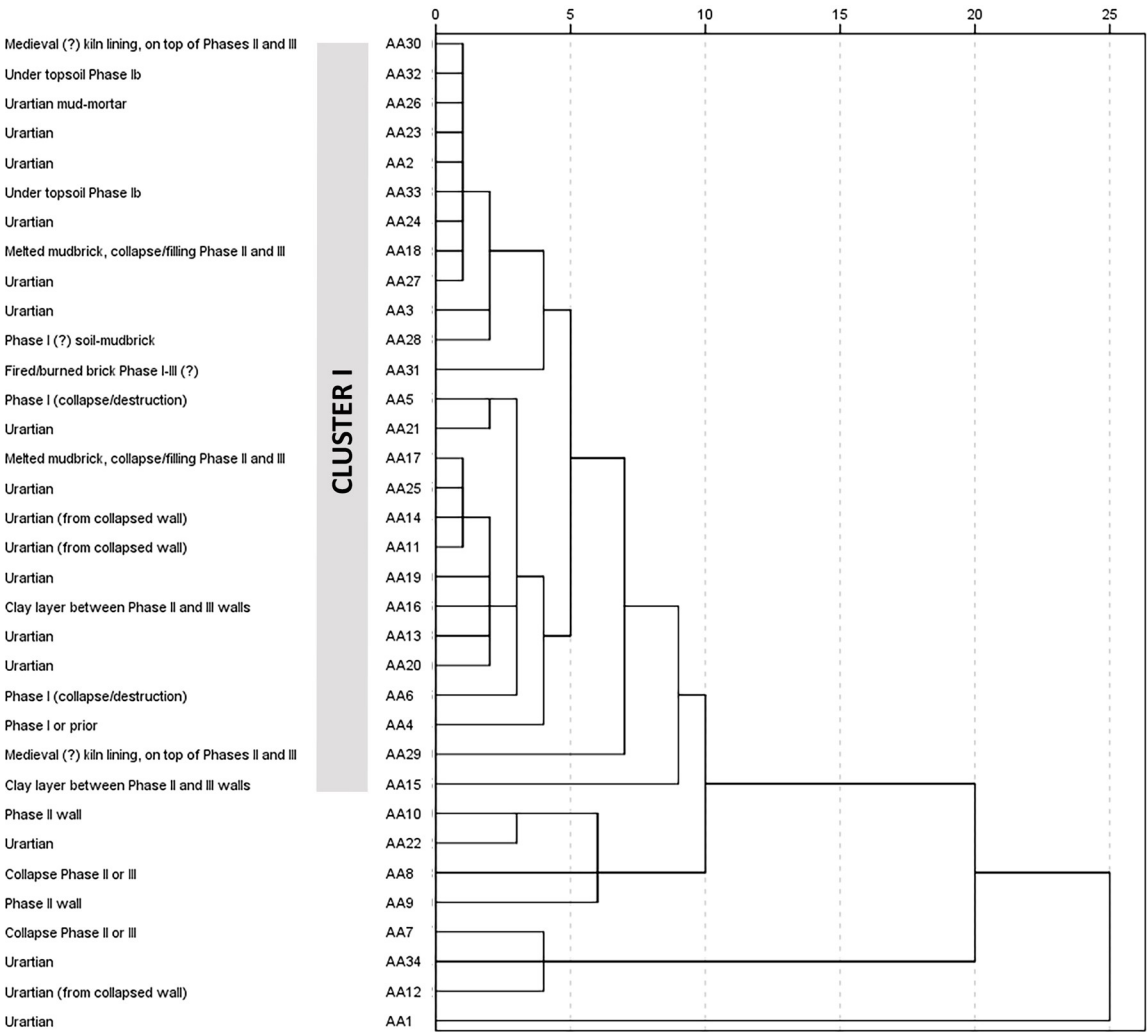
Cluster analysis dendrogram of the ED-XRF data showing one main group (Cluster 1) and outliers with centroid method based on 15 variables. Archaeological context for each sample is shown on the left side. (Credit: Elisabeth Holmqvist).

The cluster analysis dendrogram (Fig 5 and Table 3) of the ED-XRF geochemical dataset indicates one main cluster (Cluster 1), including 26 of the 34 analyzed samples, and compositional outliers which fall outside the main group. Cluster 1 is a chronologically mixed group of calcareous mudbricks (CaO c. 12.5–20 wt%), characterized by relatively high silica values (ca. 49 wt% on average) and iron values at ca.10 wt% on average. Of the eight data outliers, samples AA1, AA7, AA12 and AA34 display elevated calcium, iron, nickel, copper, and zinc oxide values; the NiO concentration of AA1 at almost 3000 ppm implies nickel contamination. Furthermore, samples AA8, AA10, and AA22 all display exceptionally high sulphur concentrations (18–24 wt%), accompanied by cobalt oxide levels at c. 120–230 ppm, potentially linking these sample materials with volcanic activity. It is also noteworthy that sample AA21 clusters with the main group, yet also shows enriched SO_3_ levels at c. 11 wt%. In addition, potential exposure to metals is suggested by the enriched nickel value of sample AA6 (NiO at c. 900 ppm), the relatively high copper content of AA17 copper (CuO c. 400 ppm), and the elevated zinc, copper and cobalt values of AA4. In addition, sample AA15 shows the highest Sr, Zr and Ba oxide concentrations measured in this sample set (Full ED-XRF results are provided in S2 File).

**Table 3:** ED-XRF results of bulk mudbrick samples.

	Phase	Na_2_O	MgO	Al_2_O_3_	SiO_2_	P_2_O_5_	SO_3_	Cl	K_2_O	CaO	TiO_2_	MnO	Fe_2_O_3_	NiO	CuO	ZnO	Rb_2_O	SrO	Y_2_O_3_	ZrO_2_	BaO	Co_2_O_3_
***Cluster 1***		%	%	%	%	%	%	%	%	%	%	%	%	ppm	ppm	ppm	ppm	ppm	ppm	ppm	ppm	ppm
AA2	Urartian	5,05	3,21	11,77	48,85	0,58	1,88	1,99	2,60	16,91	0,69	0,08	7,76	66	36	119	92	916	45	348	842	97
AA3	Urartian	2,91	2,88	11,06	48,86	0,55	0,90	0,75	2,66	19,91	1,02	0,12	9,08	112	91	67	89	1117	48	453	889	102
AA4	Hellenistic		2,55	10,34	53,98	1,81	1,90	0,80	3,11	12,47	0,97	0,29	11,42	152	114	224	130	1096	76	561	828	176
AA5	Hellenistic	2,99	2,34	9,33	44,41	1,13	3,73	0,85	3,16	20,04	1,12	0,22	11,30	364	76	177	98	1310	46	463	790	18
AA6	Hellenistic		2,11	10,06	45,68	0,63	2,62	0,81	3,16	19,86	1,17	0,17	13,19	874	93	168	139	1322	59	611	998	88
AA11	Urartian		2,94	11,26	50,49	0,90	0,43	0,06	3,23	18,04	0,84	0,20	11,28	145	87	186	135	1156	53	534	917	19
AA13	Urartian	1,47	2,98	11,16	46,77	0,48	0,26	0,04	3,30	19,69	1,22	0,27	11,98	224	80	160	145	1046	65	565	857	24
AA14	Urartian	1,47	3,19	11,89	50,49	0,50	0,33	0,05	2,93	16,40	1,01	0,16	11,17	185	99	126	129	1008	65	566	1144	51
AA15	Hellenistic		2,96	11,43	47,08	0,93	1,04	0,83	4,18	16,80	0,80	0,19	13,18	258	73	203	164	1554	36	710	2601	
AA16	Hellenistic	5,49	2,94	10,61	45,06	0,80	0,57	1,71	3,02	18,59	0,82	0,17	9,84	182	51	133	126	1475	65	594	987	54
AA17	Hellenistic	3,45	3,35	11,25	47,19	0,63	1,77	0,75	2,90	17,30	0,94	0,13	9,86	170	403	127	141	1183	52	570	814	
AA18	Hellenistic	2,52	3,23	11,73	48,31	0,57	2,24	0,54	2,91	17,23	0,77	0,14	9,53	206	47	83	93	911	55	438	788	47
AA19	Urartian	3,32	3,46	11,19	48,07	0,42	1,67	1,35	2,91	16,44	0,82	0,16	10,90	165	61	133	170	1355	73	519	986	10
AA20	Urartian	3,05	3,05	10,94	45,54	0,41	1,70	0,89	3,14	18,54	0,97	0,25	11,11	192	71	117	145	1374	87	647	1035	66
AA21	Urartian	1,70	2,68	9,41	40,44	0,37	11,02	0,61	2,47	20,61	0,93	0,18	10,34	243	95	162	109	1034	58	476	721	18
AA23	Urartian	2,70	3,45	11,82	51,55	0,78	1,05	0,49	2,72	15,66	0,87	0,13	8,51	153	61	101	92	921	50	339	679	58
AA24	Urartian	3,16	3,79	12,98	51,97	0,42	1,48	0,40	2,41	15,21	0,70	0,10	7,12	60	29	81	75	736	36	302	571	4
AA25	Urartian	3,13	3,94	12,29	48,83	0,48	1,21	0,53	2,85	16,56	0,97	0,14	9,77	143	67	116	136	1002	55	504	736	12
AA26	Urartian	2,62	3,75	13,93	51,77	0,80	1,34	0,33	2,73	14,01	0,84	0,11	7,56	93	16	89	98	716	56	311	649	
AA27	Urartian	2,27	3,71	12,28	49,98	0,51	0,44	0,27	2,80	17,27	0,93	0,18	9,78	128	60	114	98	861	65	351	687	
AA28	Hellenistic	3,52	3,77	11,87	48,81	0,85	0,59	0,55	2,63	17,49	1,14	0,17	8,06	169	70	126	100	1149	50	446	1032	22
AA29	Hellenistic	2,85	4,14	13,20	49,63	0,98	2,26	0,55	3,45	13,62	0,72	0,39	7,88	177	38	102	96	895	50	501	687	20
AA30	Hellenistic	2,20	3,77	12,83	51,20	0,88	2,71	0,47	3,05	14,48	0,75	0,14	8,04	358	29	79	78	868	50	357	604	
AA31	Hellenistic	3,56	5,09	12,48	48,69	0,33	0,58	0,42	2,54	13,09	0,84	0,18	11,90	329	77	123	63	556	47	311	397	
AA32	Hellenistic	3,06	4,08	13,29	52,29	0,41	0,55	0,23	2,78	14,33	0,74	0,15	7,82	123	45	78	86	770	47	335	705	
AA33	Hellenistic	3,47	3,78	12,26	48,88	0,87	1,12	0,80	2,67	18,27	0,71	0,08	6,80	190	30	95	90	832	32	329	564	
	μ (n = 26)	3,00	3,35	11,64	48,65	0,69	1,75	0,66	2,93	16,88	0,90	0,17	9,81	210	77	127	112	1045	55	467	866	49
	σ	0,97	0,64	1,15	2,91	0,32	2,08	0,46	0,37	2,28	0,15	0,07	1,85	156	71	41	29	249	13	118	394	44
AA1	Urartian		1,75	12,09	40,01	0,65	2,59	0,37	3,32	20,70	1,53	0,20	16,25	2908	117	367	113	883	68	398		
AA7	Hellenistic		1,29	7,43	40,56	1,10	3,16	1,38	3,11	24,47	1,30	0,26	15,96	203	101	285	193	1907	96	685	1252	
AA8	Hellenistic		1,71	6,66	26,73	0,10	24,03	0,41	2,51	25,00	1,03	0,21	11,18	188	71	144	143	1105	68	600	823	124
AA9	Hellenistic	19,91	2,22	7,39	28,69	0,51	1,13	12,95	2,63	16,51	0,56	0,13	7,04	53	38	81	93	1048	57	439	793	
AA10	Hellenistic	5,00	2,20	7,91	31,58	0,57	17,76	1,29	1,61	23,32	0,50	0,08	11,04	78		136	98	605	57	502	810	226
AA12	Urartian	1,43	1,99	9,79	45,33	0,80	0,44	0,08	3,67	19,60	1,46	0,23	15,66	299	131	239	199	1469	73	664	1090	27
AA22	Urartian		2,20	7,30	29,48	0,19	22,06	0,18	1,71	27,12	0,69	0,09	8,57	49	49	93	100	1155	54	555	615	147
AA34	Urartian	1,56	1,55	8,67	44,08	0,34	0,43	0,35	3,35	22,22	1,77	0,32	15,68	466	207	316	219	1523	96	851	1195	346

Mean values of 3–5 measurements per sample, results normalized to 100%. Cluster 1 membership and data outliers as indicated by the CA dendrogram.

### Petrographic and micromorphological results

The results of the petrographic analysis point towards one main fabric, matching the results from the geochemical analysis. This fabric is characterized by the presence of igneous rocks in a greenish-brown groundmass (Fabric 1, n = 30), with an outlier group formed by a lone individual (Fabric 2, n = 1).

Petrofabric 1 is very homogeneous in terms of geological composition, although the distribution parameters allow for a sub-classification of individuals (Sub-fabric 1.1 [c.f.v. 40:55:5]; Sub-fabric 1.2 [c.f.v. 20:75:5]; Sub-fabric 1.3 [c.f.v. 40:55:5, prominence of metamorphic rocks]) (Detailed petrographic data in the Table in S3 File). The grain size of the aplastic inclusions is generally bimodal, densely packed in the fine section and with a coarse fraction single to double spaced ranging from medium silt to granules (0.3–3 mm). The main characteristic of this petro-group is the presence of polycrystalline sub-angular to sub-rounded extrusive rocks (andesite and basalt) together with pumice fragments and greenish-yellow volcanic glass.

Aplastic inclusions also include other types of materials, but are less frequently present, in a poorly sorted grain distribution with angular to sub-rounded sphericity of the particle. It is the case of calcite, plagioclase feldspar, mono and polycrystalline quartz, metamorphic rocks (schist, phyllites), serpentine, biotite, iron-oxide nodules, pyroxenes (augite, hornblende), and rare limestone. Almost all samples of the group present a few mudstone grains of variable texture and random size, including a particular type of grey mud with chert and brown silt loam lumps. The presence of microfossils is constant and varied but not frequent. We have identified Planktonic and Benthic foraminifera, Echinoids, ostracods and gastropods. The presence of micro and mesovoids is common, but not excessively frequent, and they mainly follow the shape of vesicles or channels. The relationship between these channels and the use of vegetal degreasers is clear in the individuals AA1, 2, 3, 4, 8, 17, 20, 25, 27, 30, 31 and 34. Last, it has also been possible to identify other materials as added temper, such as charcoal in samples AA2, AA4, AA7, AA10, AA17, AA18, AA20, and AA34, sometimes reaching a large size and preserving its microstructure.

**Fig 6:**
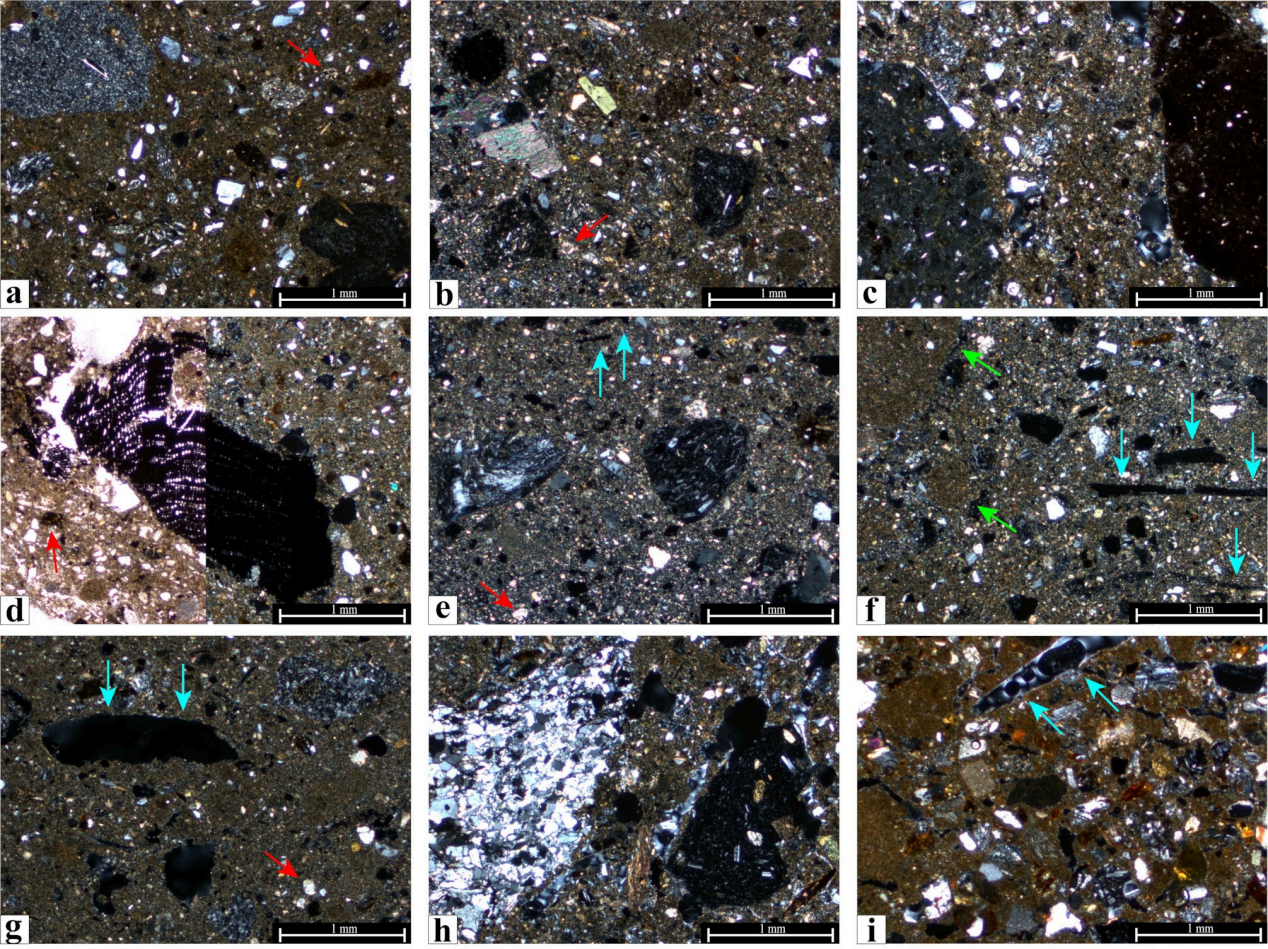
Representative photomicrographs of petrographic groups identified among the analysed mudbricks from Artaxata, crossed polars (XP). Sub-fabric 1.1: a) Urartian Sample AA21; b) Urartian Sample AA27; c) Hellenistic Sample AA10, with igneous and brown clay pellet inclusions; d) Hellenistic Sample AA18, with charcoal inclusion (Plane polarized light, PPL, and crossed polars, XP). Sub-fabric 1.2: e) Urartian Sample AA11; f) Urartian Sample AA27, showing sediment lumps pointed with green arrows; g) Hellenistic Sample AA17. Sub-Fabric 1.3: h) Hellenistic Sample AA15, showing mica schist and igneous granules. Fabric 2: i) Hellenistic Sample AA31. Red arrows point to some of the rare microfossils recognized and Blue arrows to voids linked with vegetal temper (Credit: Benjamín Cutillas-Victoria).

Concerning the internal differences within fabric 1, three subgroups can be distinguished mainly based on the frequency and grain size of the inclusions. Sub-fabric 1.1 (n = 15) includes those samples that follow the characteristics mentioned above (Fig 6A–6C), but mainly those individuals in which the coarse fraction is more noticeable, and the matrix is characterized by a high percentage of inclusions (samples AA3, 4, 5, 6, 7, 8, 9, 10, 14, 15, 16, 18, 21, 28, and 32). Sub-fabric 1.2 (n = 14) shows the same type of aplastic inclusions (Fig 6D–6F), of smaller size and in lower frequency (samples AA1, 2, 11, 12, 13, 17, 19, 20, 22, 24, 25, 26, 27, and 34). Sub-fabric 1.3 (n = 1, sample AA15) only differs in that it contains a higher presence of metamorphic rocks, mainly mica schists but also phyllite (Fig 6G).

Fabric 2 is characterized by a poorly packed brownish groundmass where the inclusions are moderately sorted (n = 1 [c.f.v. 30:60:10]) (Detailed petrographic data in S3 File). The aplastic inclusions generally reach a grain size between medium sand and silt (0.5–0.01 mm) and they are mainly represented by volcanic (andesite, basalt, volcanic glass, pumice) and metamorphic rocks (Fig 6H). We have also recognized in this sample calcite, plagioclase feldspar, quartz, serpentine, biotite, pyroxenes, and iron-oxide nodules in lesser frequency. Voids are common, although linked to two features: planar voids and vughs clearly related to the inclusion of vegetal temper, and thin channels linked with matrix microfractures.

Three thin sections were produced from sample AA34 (a-c) dated to the Urartian period and one from sample AA8 dated to the Hellenistic period. These samples are the biggest and most diagnostic samples collected in order to identify deformation features.

**Fig 7:**
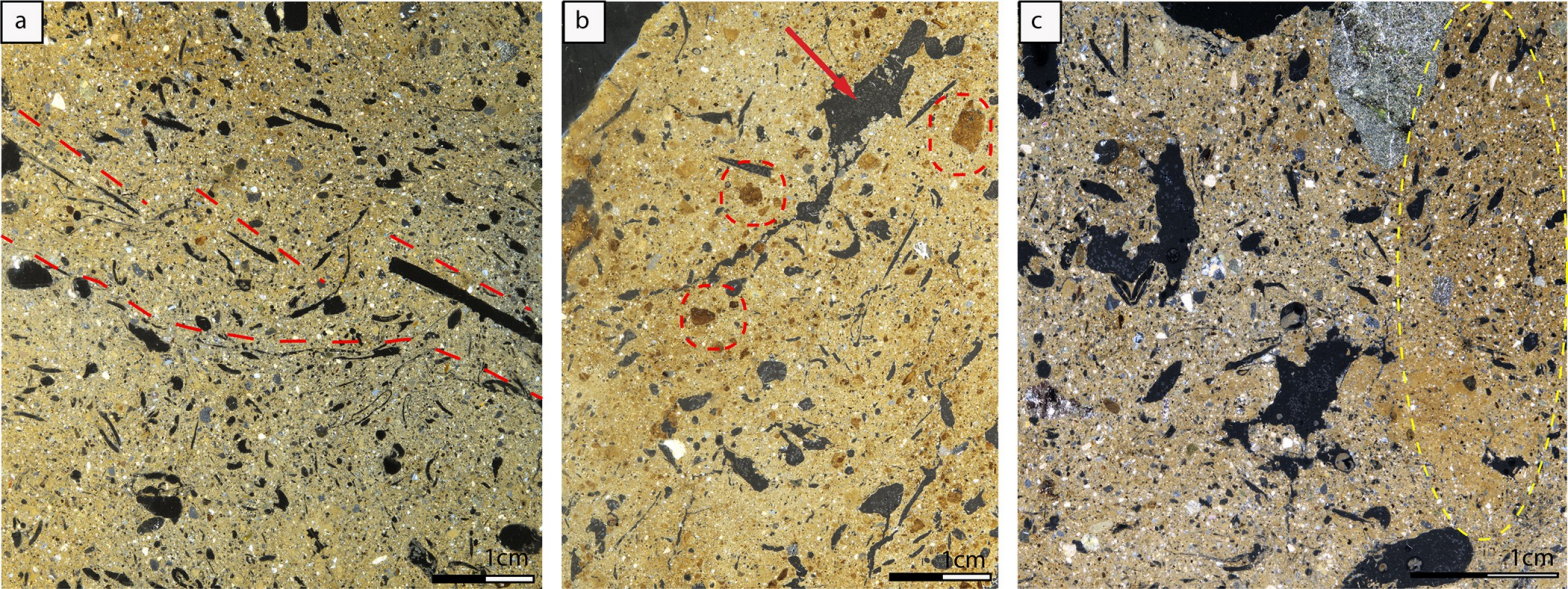
Flat scanned thin sections of thin sections AA34a and ΑΑ8 in XPL. In (a) sample AA34a, shear zones are indicated in red dotted lines in the form of elongated plant imprints and aligned vesicles. In (b) sample AA34b calcareous aggregates are indicated with a circle. Irregular shaped vugh is shown with the arrow. c) Flat scanned thin section of sample AA8 in XPL with immiscible aggregates of sediments in the circle (Credit: Mysini Gkouma).

**Fig 8:**
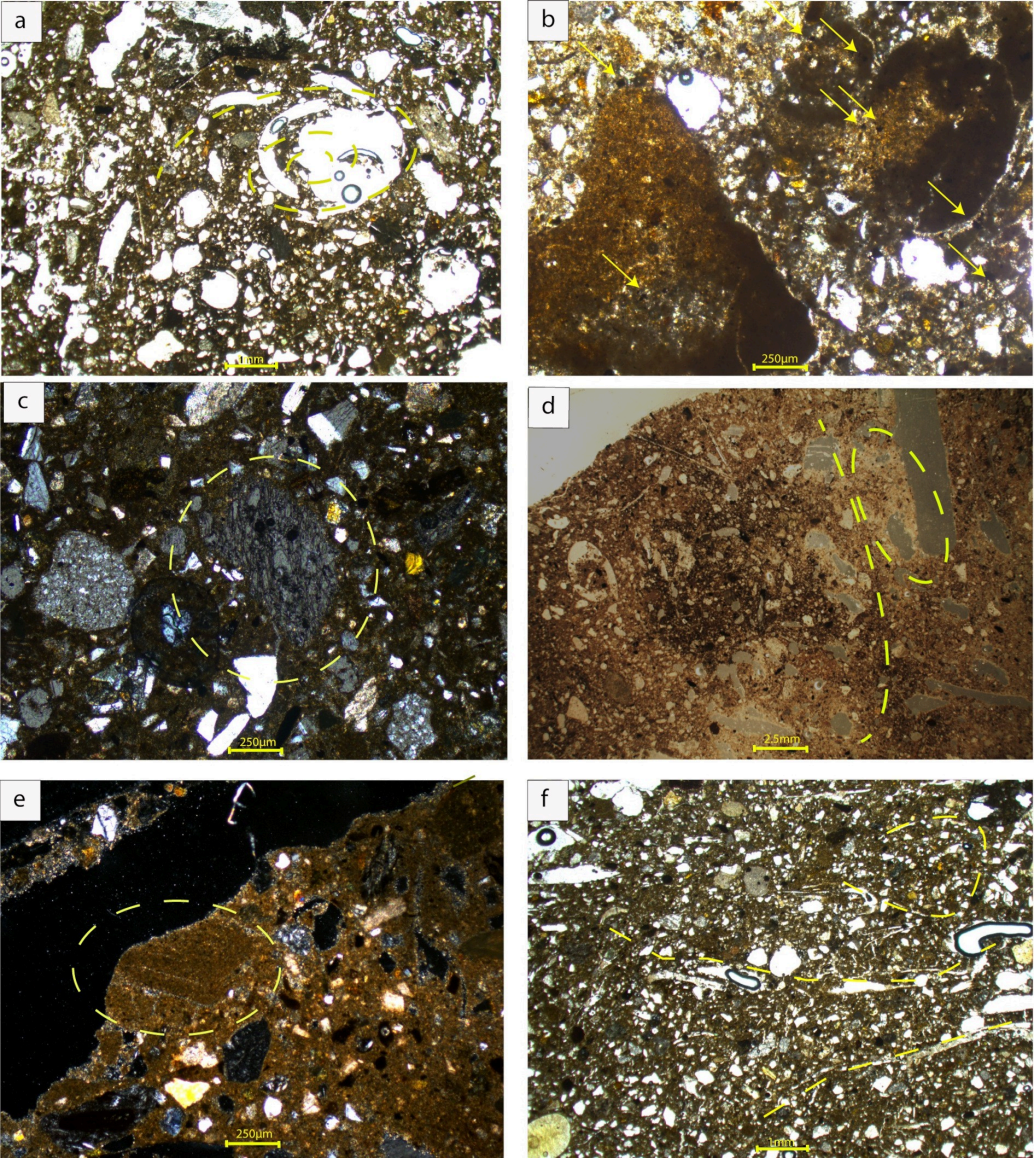
Microphotograph of AA34 (a-e) and AA8(f). a) Biopore with the spiral deformation of the plant imprint are indicated in yellow line (PPL). b) Aggregates of calcareous dusty sediments (i.e., lime nodules) with microcharcoal inclusions (PPL). c) Pumice grain in a moderately sorted matrix (XPL). d) Rounded vesicles aligned in a curvilinear axis and calcitic sediments indicative of the presence of ashes shown in the dotted circle (OIL). e) Lump of laminated sediment indicated in the yellow circle (XPL). f) Crudely aligned and rotated voids indicated in yellow dotted lines (XPL) (Credit: Mysini Gkouma).

The AA34 samples include calcareous yellowish-brown silt loams, moderately sorted with subrounded to rounded and polyconcave voids, reaching 1 cm in diameter; elongated channels are also recorded, locally curved, reflecting plant imprints of maximum 1 cm size, in irregular distribution and orientation or spiral deformation (Figs 7, 8A and 8B) (Detailed micromorphological description in S4 File). The voids are locally banded, parallel oriented and distributed, indicating shear zones (Fig 7A). Porosity is high, reaching 20%. The b/f fabric is speckled to crystallitic or undifferentiated (Fig 8D). Sand grains are sub-rounded to sub-angular and include quartz, plagioclase feldspar, chert and igneous rocks. Pumice grains are also included (Fig 8C).

Subrounded dark yellowish-brown calcareous aggregates are randomly distributed (Fig 8B). In sample AA34c these aggregates are very pronounced (Figs 7B and 8). Dispersed microcharcoal and charcoal fragments, along with calcitic sediments suggest the presence of ashes (Fig 8D).

Moreover, aggregates with laminated microstructure indicate the addition of alluvial sediments (Fig 8F).

While these results matched the initial petrographic analysis, micromorphological analysis also indicates that both the calcareous aggregates and the matrix are related to the addition of lime in the sediments. Lime is identified microscopically by the presence of a dense calcitic cementing fabric with occasionally shrinkage cracks, a few vesicles, and irregular voids with smooth walls (Fig 8B). Deformation features in these samples include the aligned and parallel oriented vughs and voids as described above, which are produced by the compaction of wet sediments when air is trapped in the voids [63]. The curved orientation of fine plant imprints further shows the direction of the shear stress while also providing clear evidence of vegetal tempering. The same pedofeature has been recognized in the Urartian Sample AA20 in thin section. Another indication of the same process is the rounded spiral deformation of plant remains, which is indicated by their imprints. In sample AA34b, deformation features are ill‐developed and limited to linear, wavy, and polyconcave voids. However, the most conspicuous feature is the presence of lime lumps in the form of poorly crystalline calcareous aggregated areas, with dark gray appearance and low birefringence, indicating only partial reaction and carbonation [64, 65].

The Hellenistic sample AA8 is largely identical to AA34. It therefore includes yellowish brown calcareous silt loams (Table in S4 File), moderately sorted with subrounded to rounded voids and elongated channels randomly distributed, locally curved, indicating plant imprints in irregular distribution and orientation (Figs 7C and 8F). The b/f fabric is speckled to crystallitic or undifferentiated. Crystallitic b/f fabric is tentatively attributed to the presence of ashes and spherulites are visible, indicating that dung was likely added to the matrix as a possible degreaser or has accidentally been included in the raw materials.

Porosity is still high (20–30%). One important novelty is the fine-grained and calcareous-lime aggregates identified in AA34, which are also recorded here although in lower proportion.

### The results of granulometric and calcimetric analyses

**Fig 9:**
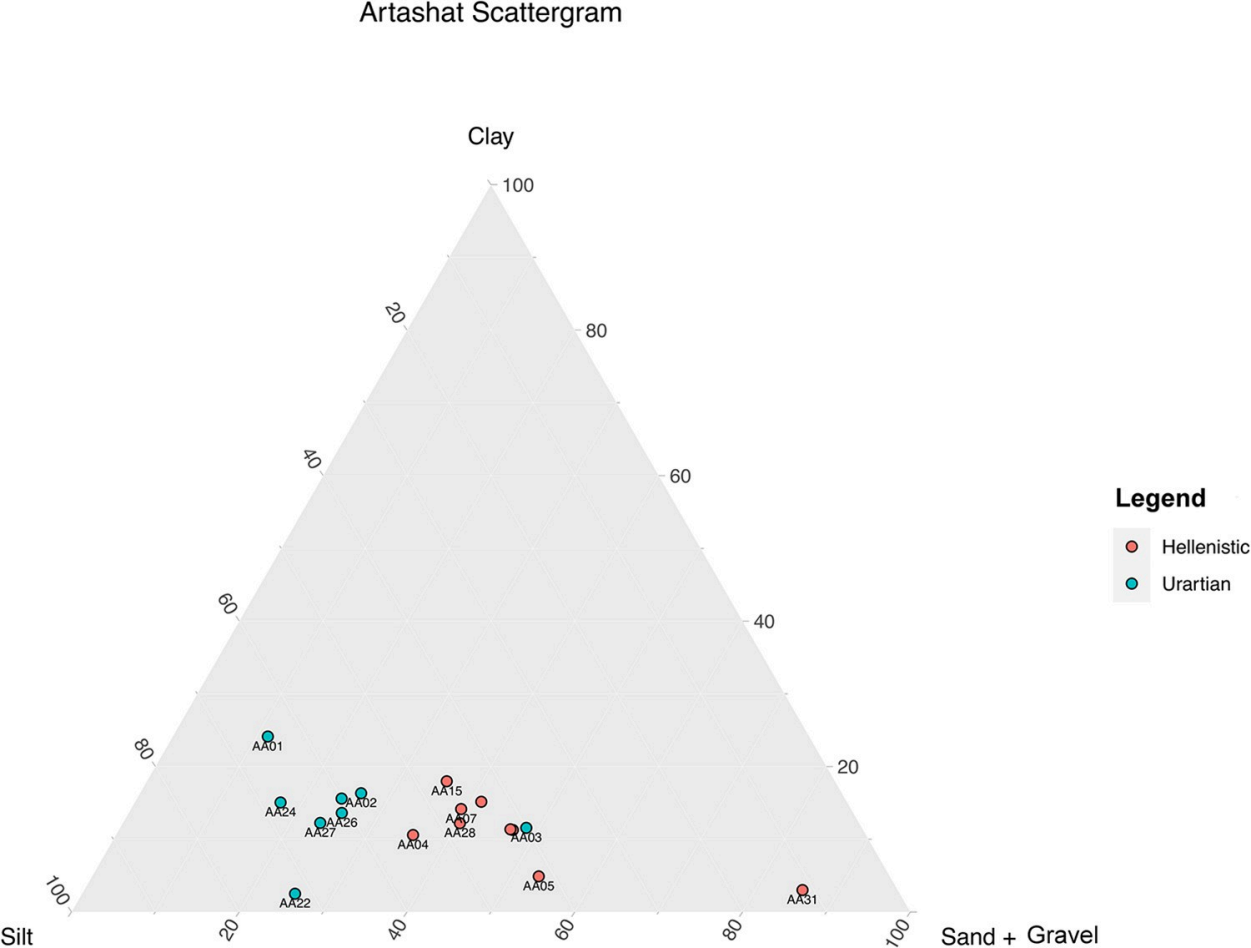
Triangular scattergram representing granulometric analysis (credit: Marta Lorenzon).

The results of the granulometric analysis suggest a slight differentiation in recipes between the Urartian and Hellenistic mudbricks (Fig 9). Urartian mudbricks seem to have a more consistent silt fraction–on average more than 50%–while Hellenistic mudbricks are characterized by a fabric with a silt fraction equal to or smaller than 40%. The organic percentage calculated through LOI is quite consistent between samples ranging between 2.5% and 5%. On the other hand, the percentage of CaCO_3_ equivalent shows variation between individuals of the Urartian and the Hellenistic period. Calcium Carbonate is higher on average in the mudbricks of the Urartian period, although its presence is also attested in Hellenistic mudbricks. This variation may be linked to the use of lime as a secondary human-induced temper alongside chaff in Artaxata mudbrick production (Table 4).

**Table 4:** Results of granulometry, LOI and calcimetry.

Sample	Area	Period	Gravel	Sand	Silt	Clay	CaCO_3_	Organic
**AA01**	Hill II	Urartian	0.18	11.17	64.54	24.11	5	5.03
**AA02**	Hill II	Urartian	1.00	25.37	57.32	16.31	11	4.76
**AA03**	Hill II	Urartian	0.70	47.74	40.01	11.55	8	3.61
**AA14**	Trench 11	Urartian	1.14	23.27	60.01	15.58	10	3.48
**AA22**	Hill II	Urartian	0.48	24.92	72.11	2.49	14	3.63
**AA24**	Hill II	Urartian	0.00	17.40	67.56	15.04	14	4.99
**AA26**	Hill II	Urartian	2.66	22.76	60.98	13.60	10	4.64
**AA27**	Hill II	Urartian	0.52	23.02	64.23	12.23	10	4.11
**AA04**	Trench 3	Hellenistic	0.98	34.45	54.00	10.57	8	2.99
**AA05**	Trench 11	Hellenistic	3.50	49.76	41.86	4.88	8	2.50
**AA07**	Trench 2	Hellenistic	0.90	38.48	46.48	14.14	8	4.40
**AA08**	Trench 1	Hellenistic	0.50	40.78	43.58	15.14	12	3.68
**AA09**	Trench 4	Hellenistic	2.58	44.40	41.76	11.26	8	3.89
**AA10**	Trench 3	Hellenistic	7.64	39.01	42.00	11.35	7	2.96
**AA15**	Trench 4	Hellenistic	5.00	30.75	46.29	17.96	10	4.75
**AA28**	SO1	Hellenistic	1.38	38.82	47.61	12.19	10	3.50
**AA31**	Trench 6	Hellenistic	1.04	84.60	11.36	3.00	5	3.31

**Fig 10:**
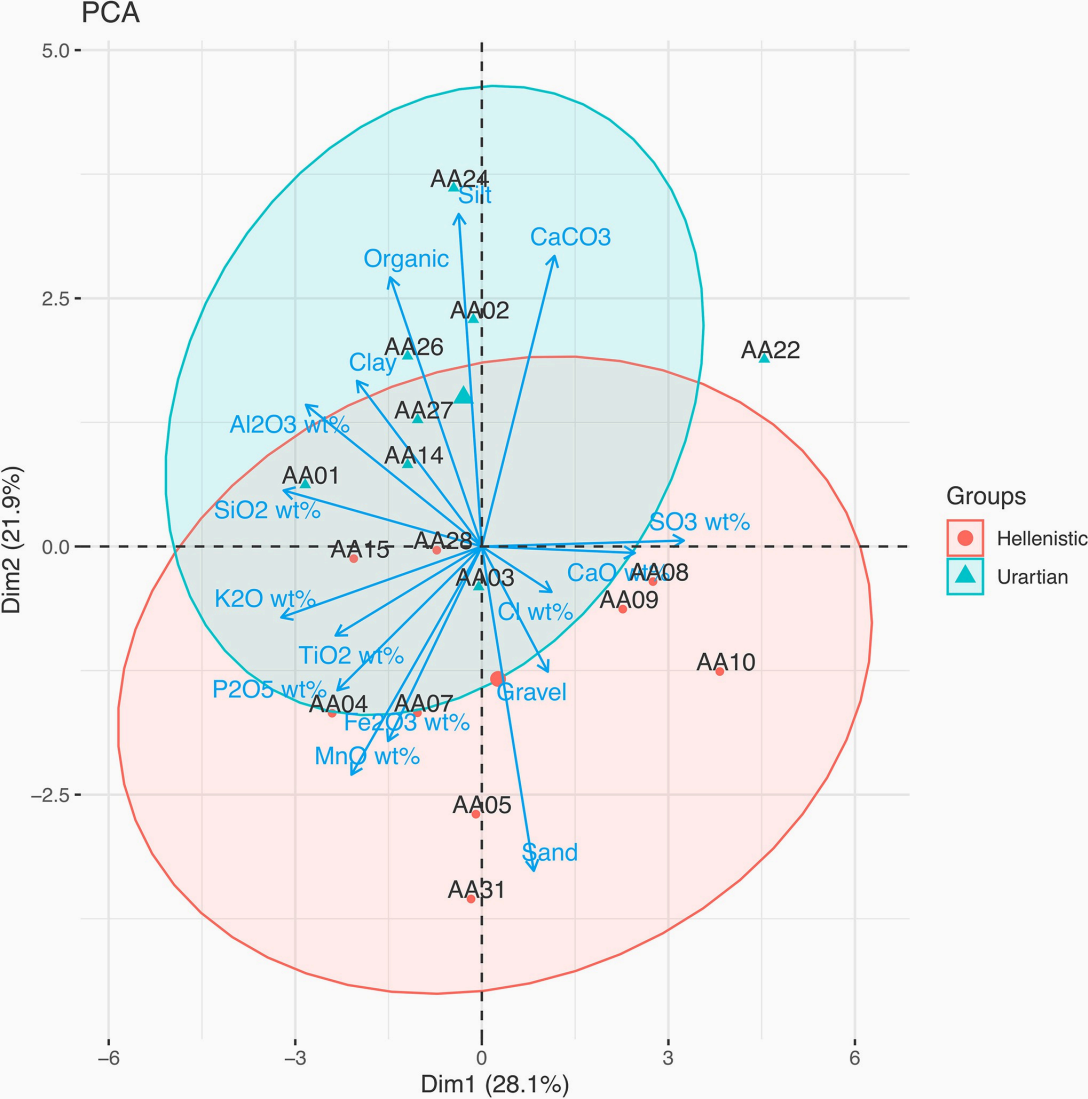
PCA mapping of the Artaxata samples based on the variables visible in the arrows. The large blue triangle and red circle represents the average value of each cluster (Credit: Marta Lorenzon).

**Fig 11:**
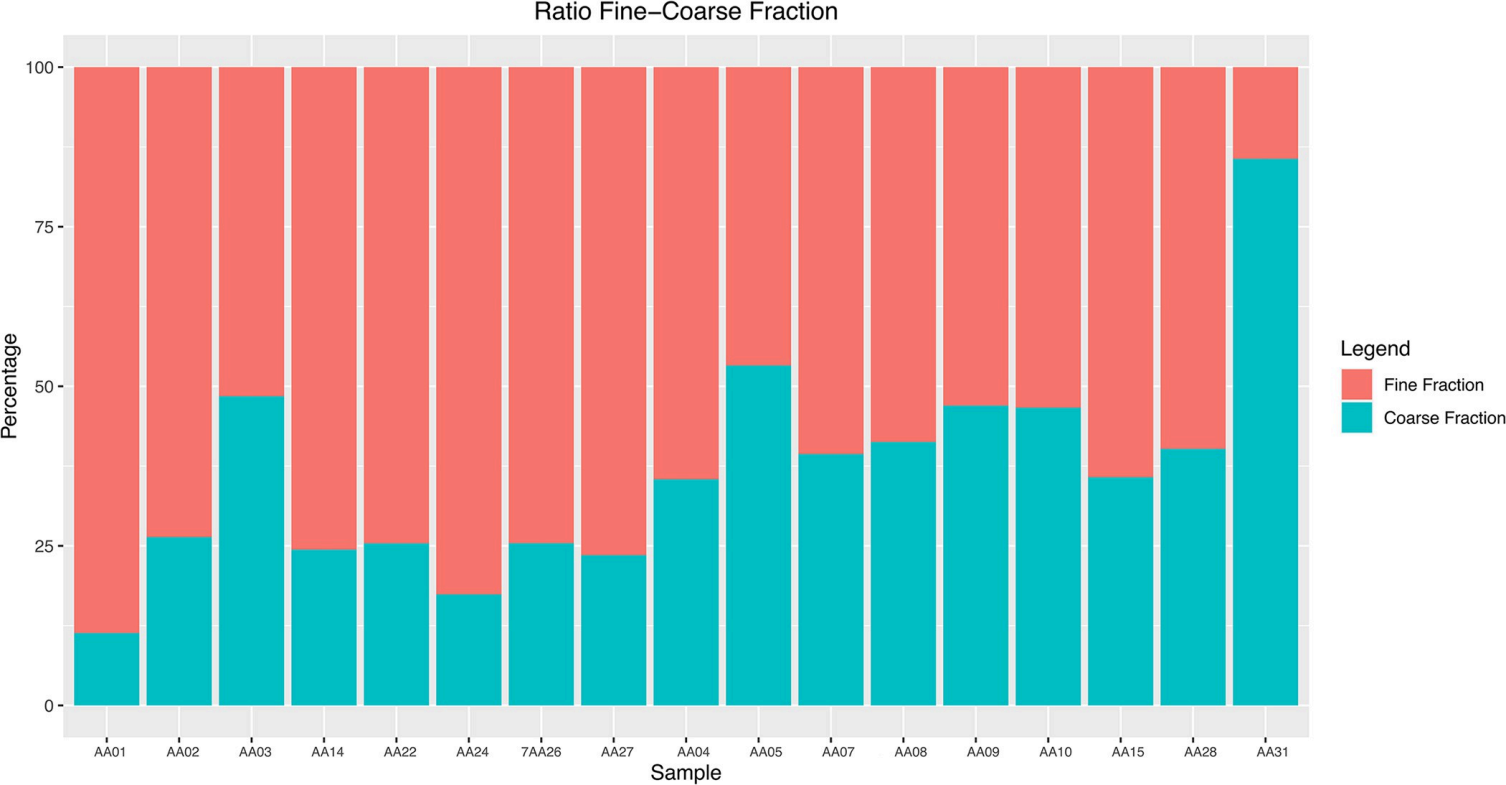
Fine and Coarse fractions in the Artaxata samples (credit: Marta Lorenzon).

The PCA combining granulometry, Organic %, CaCO_3_% and selected geochemical data (Al_2_O_3_, SiO_2_, P_2_O_5_, SO_3_, Cl, K_2_O, CaO, TiO_2_, MnO and Fe_2_O_3_ wt%) provide us with two different clusterings, highlighted by the confidence ellipses created by coding a multivariate t-distribution. The two ellipses underline a general communality of the raw sources but slightly different recipes between the Urartian and Hellenistic periods (Fig 10). The main difference, as also confirmed by the micromorphological and petrographic analysis, is the ratio variation between the fine and coarse fractions (Fig 11).

### The results of Phytolith analysis

The petrographic thin sections AA7, AA17, AA27, and AA34 were selected for exploratory analysis of the phytolith content. They represent a combination of Urartian and Hellenistic mudbricks from the two main occupation periods. Table 5 documents the absolute number of phytoliths captured by each of the observed distribution patterns in the fabric (Illustrations of the observed distribution patterns are provided in the tables in S5 File).

**Table 5:** Quantitative inventory of the phytoliths composing each of the distribution patterns observed in the clay fabric.

	ISO	CLU	ART	tot	Div
**AA 7**	31	15	3	49	9
**AA 17**	9	10		19	5
**AA 27**	10	4		14	6
**AA34**	13			13	4

ISO: Isolated; CLU: Clustered; ART: Articulated; tot: total number of phytoliths; Div: morphological diversity.

**Fig 12:**
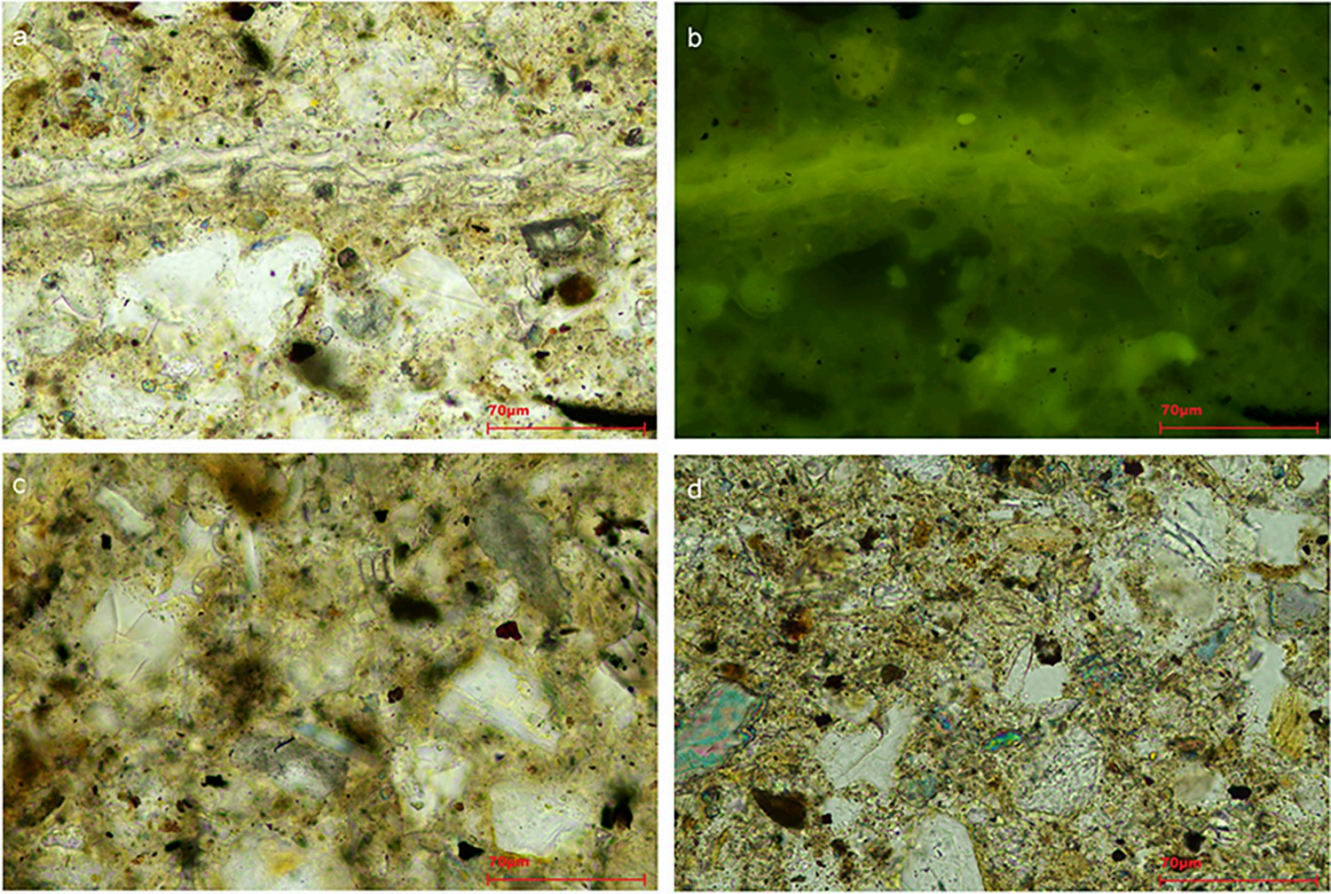
a) Silica skeleton of a phytoliths observed in the clay fabric of AA7. Stomata are the major type of phytolith composing this system. ×500; PPL. b) Silica skeleton in the clay fabric of AA7 under Blue light. The black dots correspond to the stomata. ×500; Blue. c) Fragmented diatom observed for AA 7. X500; PPL; d) Diatom frustule observed for AA17; ×500; PPL (Credit: Luc Vrydaghs).

By associating three distribution patterns (isolated, clustered and articulated) together with a more marked morphological diversity and the largest number of phytoliths, the fabric of AA7 differs from all the others. Basically, the AA7 fabric attests the richest and most diverse phytolith content. A silica skeleton was also recorded within it (Fig 12A and 12B).

The other fabrics provide additional specific features. AA17 is characterized by an association of isolated and clustered phytoliths where each pattern captures an almost equivalent number of phytoliths (of 9 [Isolated] and 10 [Clustered] respectively). Only isolated phytoliths were observed in the AA34 fabric (Table 6).

**Table 6:** Phytolith assemblages according to the distribution patterns.

			GSSCP			Elongate				Others				Div
		BIL	RON	SAD	TRZ	ELO_ENT	ELO_ENT_1	ELO_ENT_2	ELO_DET	BLO	BUL	TRA	UNK	
**ISO**	**AA 7**	X	X	X		X	X	X	X	X	X		X	10
	**AA 17**		X					X			X			3
	**AA 27**		X		X			X	X					4
	**AA 34**		X			X	X	X						4
**CLU**	**AA 7**		X			X	X							3
	**AA 17**						X	X						2
	**AA 27**		X				X					X	X	4
	**AA 34**													

ISO: Isolated; CLU: Clustered; GSSCP: Grass Silica Short Cells. BIL: Bilobate; RON: Rondel; TRZ: Trapezoid. Elongate: ELO_ENT: Elongate entire; ELO_ENT_1: Elongate entire 1; ELO_ENT_2: Elongate entire 2; ELO_DET: Elongate dentate. Other: BLO: Blocky; BUL: Bulliform; TRA: Tracheary; UNK: Unknown. No Clusters were observed for AA34.

Analyzing the petrofabrics, we noted the presence of numerous phytoliths and diatoms. While phytoliths were present in all the analyzed thin sections, diatoms were not observed for AA34 but were for AA7, AA17, and AA27 (Fig 12C and 12D).

Even considering the limited number of thin sections analyzed, the AA7 petrofabric appears to be the one richest in phytoliths, the total number of observed phytoliths being at least 2.5 times greater than any others.

**Fig 13:**
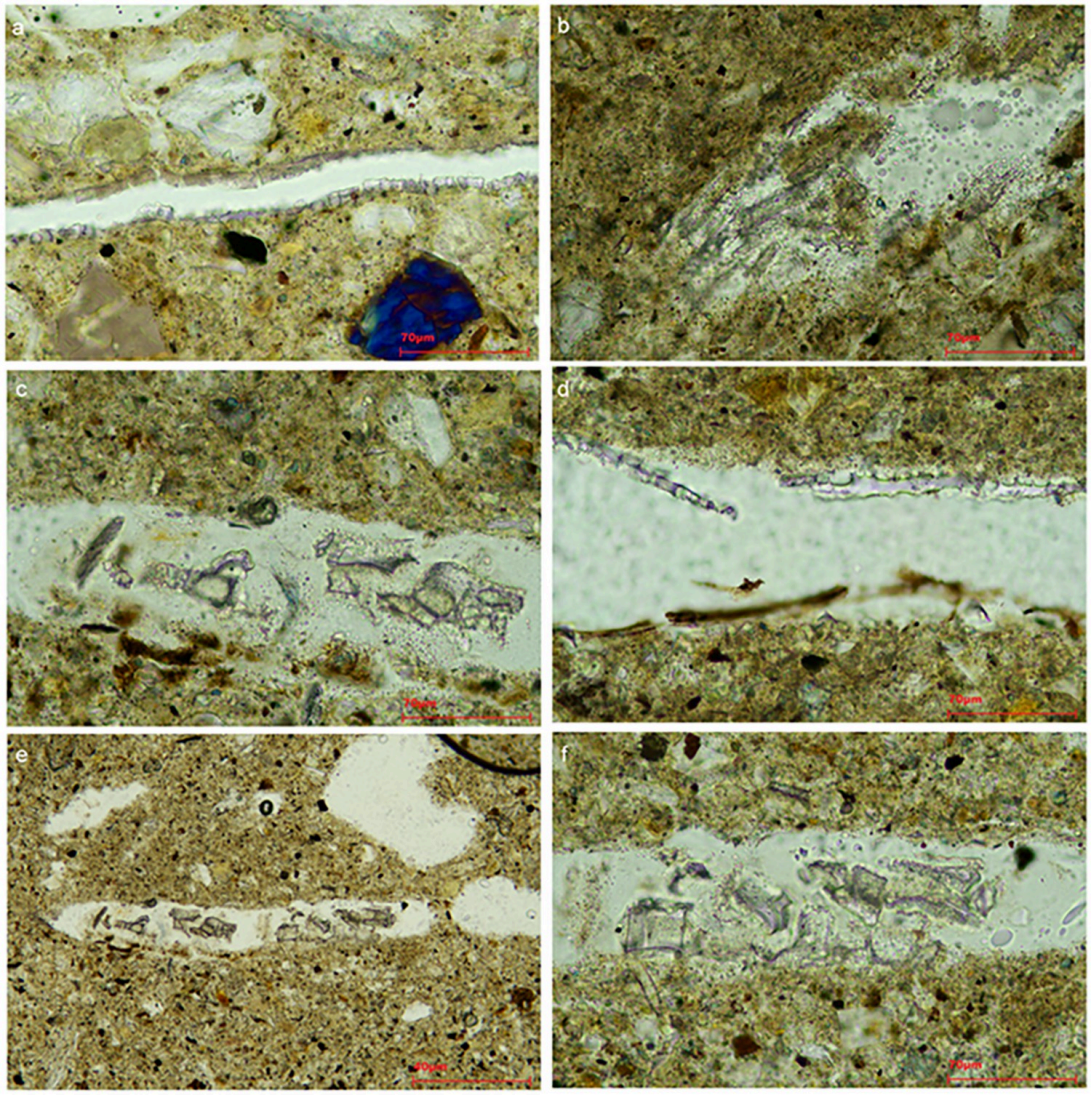
Phytoliths in sample AA17, ×500, PPL. a) Elongate dentate/dendritic observed in almost side view along the limit of the void; b) Clusters of different types of phytoliths (among which Blocky) observed in the light of a void; c) Elongate dentate/dendritic observed in almost side view along the limit of the void; d) Elongate dentate/dendritic observed in almost side view along the limit of the void; e) Clusters of unidentified phytoliths observed in the light of a void; f) Second cluster of phytoliths observed in the light of a void. On the extreme left, a phytolith named as Blocky (Credit: Luc Vrydaghs).

Concerning other differences between the samples, phytoliths in voids were distinctly observed only for AA17 (Fig 13). Their distribution pattern (clustered or articulated) and inventory differ according to the considered void. Elongate dentate are distributed as they are when in anatomical position (Fig 13A, 13C and 13D). In other voids, phytoliths are observed in clusters (Fig 13E and 13F). Some can be named as Blocky (Fig 13F). Elongate dentate (Fig 13A, 13C and 13D) derive from the inflorescence bract of cereals [61].

## Discussion

The combination of results provides us with a new perspective on human-environment interaction in Artaxata during the 1st millennium BC, specifically during the Hellenistic period. The chemical and petrographic data point to a local source for procurement of raw materials for earthen building materials. Soil resources can easily be identified within walking distance from the site, including the geological deposits located within a radius of a few km from the site (Fig 3). For instance, the alluvial deposit that constitutes the backbone of Artaxata geology is the main source of soil procurement strongly influenced by the volcanic materials north of the settlement and from nearby Mount Ararat, which are characteristic of the matrix inclusions and aggregates in all of the mudbricks manufactured at Artaxata. The presence of polycrystalline extrusive rocks such as andesite and basalt in petrofabric 1 together with pumice fragments and greenish-yellow volcanic glass perfectly matches up with the geological characteristics of Artaxata’s environment, very conditioned by the igneous materials from Mount Ararat [66, 67].

On the other hand, the ratio of clay, silt and sand, carbonates and the phytolith inventory provide indications of multiple manufacturing phases and likely material re-use.

The PCA mapping of the granulometric analysis, organic percentage, chemical compounds and CaCO_3_ equivalent highlights the presence of two distinct clusters (Fig 10). When we incorporate the analysis of the grain size and the matrix typology in thin sections, we noted a change of the mudbrick recipes between the Urartian and Hellenistic periods. Although some Hellenistic samples have been classified in the coarser matrix Sub-Fabric 1.1., a thinning of the recipe, which means a smaller fine fraction, characterized a significant part of the Hellenistic mudbricks and could be clearly associated with cyclical re-use of earthen building materials found at the site by the Hellenistic communities that worked on mudbrick manufacturing. The collection of new raw materials is often a time-consuming and economic burden for manufacturers [68]; thus, the presence of abandoned structures at the site could provide the necessary material while removing a consistent chunk of the traditional *chaîne opératoire* [14]. To test this hypothesis, we created multiple micromorphological sections to analyze the morphological differences between mudbricks of different periods.

The micromorphological studies support our initial hypothesis, identifying the aggregates in the Urartian sample AA34 as immiscible, suggesting the incomplete mixture of diverse materials (Fig 8B). In a few cases, sediments are characterized by a laminated microstructure, indicating their alluvial origin (Fig 8E) and matching the geological deposits around Artaxata. More often the Urartian sample AA34 includes fine-grained and calcareous-lime aggregates, as described above.

This observation is tentatively interpreted as correlating well with the results of granulometry and LOI. More specifically, the lower percentages of silts and CaCO_3_ in AA8 possibly reflect the lower number of fine-grained lime inclusions in comparison to AA34. The deformation features are here limited and ill developed with abundant polyconcave vughs. The matrix is crystallitic due to the presence of ashes and spherulites are visible, indicating that dung was likely added to the matrix as a possible degreaser or has accidentally been included in the raw materials. The presence of pedomorphic voids and the constant organic percentage indicates that organic matter was re-added during the Hellenistic manufacture and mixed with water and the broken-down mudbricks, a key source of appropriate mudbrick sediments.

In line with this argument, the presence of immiscible aggregates of sediments in Hellenistic sample AA8, may be associated with fragments of re-used broken-down mudbricks (Fig 7C), which presented characteristics clearly in line with the petrography of Urartian mudbricks (i.e., Vegetal temper and mineral inclusions), and stands out in the Hellenistic mudbrick matrix as not completely integrated into the new structure.

The data presented and the difference in morphological and grain size percentage between Urartian and Hellenistic samples seems to point to a specific process of re-use of building materials by Hellenistic communities. Urartian mudbricks standing in situ had progressively deteriorated due to abandonment, wind erosion, water erosion, temperature related deterioration, and chemical related deterioration [69, 70], leading to a loss of the fine fraction in the bricks. While wind erosion tends to affect the coarse fraction within a mudbrick more, the other three types of deterioration affect the fine fraction equally. For instance, AA9 shows anomalously high sodium and chloride oxide levels at c. 20 wt% and c. 13 wt%, respectively, indicating possible salty water exposure or sodium chlorite mineral content. At Artaxata, the high values of soluble substances such as Na^+^, S, Cl^-^ and Ca in the soil combined with the high porosity of the mudbricks and the extensive presence of water that permeated the bricks over time, such as in the case of precipitation, generated both water and chemical related erosion, thus deeply affecting the structural integrity and the fine fraction of the bricks [18].

A similar phenomenon is observed in many earthen structures when left exposed without continuous maintenance [18, 70, 71]. Mudbrick left exposed undergoes rapid deterioration that especially affects the fine fraction due to the loss of material [19, 31], but also the cementing of smaller grains by salt crystallization such as calcium carbonate [71].

Once these were broken down and re-molded during the Hellenistic period, there was little to no addition of new sediments, mainly supplementing the recipe with new vegetal temper and other aggregates. This explains the same approximate percentage of organic temper and CaCO_3_ between Hellenistic and Urartian mudbricks, but phytoliths clearly visible within voids were recorded only for AA17. This implies that plant material was used as temper. Their inventory tends to support the use of diverse plant material alongside by-products of cereal processing. The taxonomical occurrences of Blockies are diverse and very common in the leaves of Cyperaceae and Poaceae as well as in dicots and conifers [61]. Hence, one cannot exclude the use of diverse types of plant material as temper, among which is the by-product of cereal processing.

Urartian mudbricks tend to show a consistently denser structure with a few thin elongated features where fibrous vegetal temper was present, as well as rotatory features as a result of the extensive kneading, On the other hand, Hellenistic mudbricks are characterized by a less dense structure with crudely aligned and rotated voids as evidence of limited tactile manipulation [14], in which the incorporation of previous EBM may be detectable.

Later processes could determine the phytolith content (distribution patterns, phytolith amounts and morphotypes) of the Hellenistic mudbricks. The phytolith inventory reveals that almost all phytoliths distributed along the Isolated and Clustered patterns are clustered within the Hellenistic petrofabric (Table 5). Clusters consist of a group of disarticulated phytoliths where not all phytoliths are necessarily of the same type or share the same orientation (Figs 12 and 13). These are understood as typical markers of post-depositional perturbations resulting from the physical reworking of the original material [59]. As such, the clusters observed for the Hellenistic mudbricks, as the studied material allows us to characterize them, could result from the re-use of Urartian mudbricks.

Granulometry, specifically the measurement of the ratio of clay-silt and sand, remains one of the most reliable variables in the analysis and classification of mudbrick recipes [4, 6, 12], but both micromorphology and phytolith analysis reveal themselves as key methodological additions to enhance our ability to discern cases of re-use. The new methodology employed, specifically the phytholiths’ analysis and micromorphology, allows us to better understand the relationship between the natural and built environment, raw source-material procurement choices in the Hellenistic period, and the intensity of the construction process.

The recycling of mudbrick in architecture is not a new phenomenon [4, 72], but in most cases the argument for re-use discusses the removal of finished artifacts, in this case bricks, from ancient walls. In our argument we discussed a different phenomenon in which previous building materials are re-used as an available and ready-made source of soil. This shortcut alters the traditional relationship between communities and the environment, indicating the opportunistic nature of Artaxata Hellenistic communities regarding mudbrick manufacturing and the *chaîne opératoire*. This may have been prompted by the need for a fast construction process and the necessity to build a new center in a short period of time.

Additionally, the benefit of using already existent earthen building materials stresses the rational use of sources of raw materials within an almost carbon-neutral construction process that cut the energy inputs, such as carrying the raw material up a steep hill, to maximize efficiency.

The evidence supports the written sources that, at the time of Artaxias I’s foundation in the 180s BC, the previous settlement had been abandoned for quite some time and the previous structures had deteriorated. Since Artaxata was not continuously inhabited during the 1st millennium BC this seems to indicate that, during Hellenistic Phase I and II/III, the community re-used abandoned and exposed EBM in order to have economical and advantageous access to soil sources for their constructions.

## Conclusions

Our research built upon previous mudbrick research, emphasizing the relevance of geochemistry and grain size analysis in the study of mudbrick, but also provide new evidence on the methodological importance of micromorphology and phytolith analysis to discern cases of earthen building material re-use. The data clearly indicated that an interdisciplinary approach is better suited to investigate the complex relationship between the architecture, natural environment, and past communities.

Finally, the recycling of earthen building materials is a well attested phenomenon that is often difficult to attest in the archaeological record. Our research presents an innovative methodology to observe reusing of mudbricks in archaeological sites employing geochemistry, micromorphology and phytoliths analysis. The method is relevant for archaeological and architectural studies, specifically those dealing with mudbrick architecture.

## Supporting information

S1 File 14C dating parameters. Supplementary information in relation to 14C dating.
(DOCX)

S2 File ED-XRF table. The table presents all measured elements with standard deviation and relative standard deviation.
(XLSX)

S3 File Petrographic table. Summary of the petrographic fabrics identified in Artaxata.
(DOCX)

S4 File Micromorphology table. Summary of the micromorphological analysis in Artaxata.
(DOCX)

S5 File Phytoliths tables. Supplementary information from phytoliths analysis: Table 1. Illustrations of Distribution Patterns (1.1 Isolated and 1.2 Clustered); Table 2. Illustration of phytoliths observed in the clay fabric.
(DOCX)

## References

[1] Baudouin E. Spread and Independent Technical Invention of the Earthen Material in the Southern Caucasus and Northern Mesopotamia during the sixth Millennium BCE. In: Daneels A, Torras Freixa M, editors. Earthen construction technology. Oxford: Archaeopress; 2021. p. 15–54.

[2] Hansen S, Mirtskhulava G, Bastert-Lamprichs K. Aruchlo: a Neolithic settlement mound in the Caucasus. Neo-Lithics. 2007; 1(7): 13–19.

[3] Nishiaki Y, Guliyev F, Kadowaki S, Omori T. Neolithic residential patterns in the southern Caucasus: Radiocarbon analysis of rebuilding cycles of mudbrick architecture at Göytepe, West Azerbaijan. Quat Int. 2018; 474: 119–130. 10.1016/j.quaint.2017.09.015

[4] Rosenberg D, Love S, Hubbard E, Klimscha F. 7,200 years old constructions and mudbrick technology: The evidence from Tel Tsaf, Jordan Valley, Israel. PloS ONE. 2020; 15(1): e0227288. doi: 10.1371/journal.pone.0227288 31968007 PMC6975557

[5] Lorenzon M. From chaff to seagrass: The unique quality of Minoan mudbricks. A geoarchaeological approach to the study of architectural craft specialization in Bronze Age Crete. J Archaeol Sci Rep. 2021; 40: e103122. 10.1016/j.jasrep.2021.103122

[6] Love S. An archaeology of mudbrick houses from Çatalhöyük. In: Hodder I. editor. Substantive Technologies at Çatalhöyük: Reports from the 2000–08 Seasons. Los Angeles: Cotsen Institute of Archaeology; 2013. p. 81–96.

[7] Azzazy MF. Exploratory palynological studies at the Tell el-Daba’a-Avaris archaeological site. PloS ONE. 2018; 13(2): e0180770. doi: 10.1371/journal.pone.0180770 29415021 PMC5802438

[8] Devolder M, Lorenzon M. Minoan master builders? A diachronic study of mudbrick architecture in the Bronze Age Palace at Malia (Crete). Bull Corresp Hell. 2019; 143(1): 63–123.

[9] Khachatryan Z.D. Artašat II. antik dambaranadaštə (1971–1977 t‘t‘. pełumnerə). Yerevan; 1981.

[10] Arakelian BN. Artashat I. Yerevan; 1982.

[11] Invernizzi A. Ai piedi dell’Ararat. Artaxata e l’Armenia Ellenistico-Romana Invernizzi. Firenze: Le Lettere; 1998.

[12] Lorenzon M, Nitschke JL, Littman RJ, Silverstein JE. Mudbricks, construction methods and stratigraphic analysis: a case study at Tell Timai (Ancient Thmuis) in the Egyptian Delta. Am Archaeol J. 2020; 124(1): 105–131. doi: 10.3764/aja.124.1.0105

[13] Wright GRH. Ancient Building Technology, Volume 2: Materials. Leiden: Brill; 2005.

[14] Lorenzon M. Earthen Architecture as a Community of Practice: A Case Study of Neolithic Earthen Production in the Eastern Mediterranean. Camb Archaeol J. 2023; First View: 1–18. 10.1017/S0959774323000033

[15] Goldberg P. Geology of Late Bronze Age mudbrick from Tel Lachish. Tel Aviv. 1979; 6(1–2): 60–69. 10.1179/033443579788497478

[16] Lorenzon M, Iacovou M. The Palaepaphos-Laona rampart: A pilot study on earthen architecture and construction technology in Cyprus. J Archaeol Sci Rep. 2018; 23: 348–361. 10.1016/j.jasrep.2018.11.004

[17] Prévost‑Dermarkar S. Bâtir en terre au Néolithique. Approche morpho-technologique des vestiges architecturaux de Dikili Tash (Grèce). Bull Corresp Hell. 2019; 143(1): 1–61.

[18] Friesem DE, Boaretto E, Eliyahu-Behar A, Shahack-Gross R. Degradation of mud brick houses in an arid environment: a geoarchaeological model. J Archaeol Sci. 2011; 38(5): 1135–1147. 10.1016/j.jas.2010.12.011

[19] Goodman-Elgar M. The devolution of mudbrick: ethnoarchaeology of abandoned earthen dwellings in the Bolivian Andes. J Archaeol Sci. 2008; 35(12): 3057–3071. 10.1016/j.jas.2008.05.015

[20] McIntosh SK, MacDonald KC. Sponge Spicule in Pottery: New Data from Mali. J Field Archaeol. 1989; 16: 489–494. 10.1179/jfa.1989.16.4.489

[21] Goren Y, Goldberg P. Special Studies: Petrographic Thin Sections and the Development of Neolithic Plaster Production in Northern Israel. J Field Archaeol. 1991; 18(1): 131–140. 10.1179/009346991791548735

[22] De Paepe P, Rutten K, Vrydaghs L, Haerinck E. Petrographic, chemical and phytolith analysis of late pre-islamic ceramic from ed-Dur, Um el-Kawein (U.A.E.). In: Potts D, Hasal Al Naboodah H, Hellyer P, editors. Archaeology of the United Arab Emirates. Proceedings of the First International Conference on the Archaeology of the United Arab Emirates. London: Trident Press Ltd.; 2003. p. 81–96.

[23] Starnini E, Szakmány G, Madella M. Archaeometry of the first pottery production in the Carpathian Basin: results from two years of research. In: D’Amico C, editor. Atti Del IV Congresso Nazionale Aiar. Pisa, 1–3 febbraio 2006. Bologna: Pátron Editore; 2007. p. 401–411.

[24] Lippi MM, Gonnelli T, Pallecchi P. Rice chaff in ceramics from the archaeological site of Sumhuram (Dhofar, Southern Oman). J Archaeol Sci. 2011; 38: 1173–1179. 10.1016/j.jas.2010.09.028

[25] Tomber R, Cartwright C, Gupta S. Rice temper: technological solutions and source identification in the Indian Ocean. J Archaeol Sci. 2011; 38(2): 360–366. 10.1016/j.jas.2010.09.014

[26] Kreiter A, Pető Á, Pánczél P. Materializing tradition: ceramic production in Early and Middle Neolithic Hungary. In: Bánffy E, editor. The Early Neolithic of the Danube-Tisza Interfluve. Oxford: Archaeopress; 2013. p. 127–140.

[27] Kreiter A, Riebe DJ, Parkinson WA, Pető Á, Tóth M, Pánczél P. Unique in its Chaîne Opératoire, Unique in its Symbolism: Undressing a Figurine from the 6th Millennium BC Körös culture, Hungary. J Archaeol Sci. 2014; 44: 136–147. 10.1016/j.jas.2014.01.027

[28] Chowdbury KA, Ghosh SS. Plant remains from Hastinapura. AncInd. 1954; 10: 120–137.

[29] Stoops G, Nijs R. Micromorphological characteristics of some tell materials from Mesopotamia. Pedologie. 1986; 3: 329–336.

[30] Delhon C. Potentiel de l’analyse des phytolithes contenus dans les pâtes céramiques et les matériaux de construction. Cahier des thèmes transversaux ArScAn. 2005/06; 7: 86–93.

[31] Friesem DE, Tsartsidou G, Karkanas P, Shahack-Gross R. Where are the roofs? A geoethnoarchaeological study of mud brick structures and their collapse processes, focusing on the identification of roofs. Archaeol Anthropol Sci. 2014; 6(1): 73–92. 10.1007/s12520-013-0146-3

[32] Ryan P. Plant as material culture in the Near Eastern Neolithic: perspectives from the silica skeleton artefactual remains at Çatalhöyük. J Anthropol Archaeol. 2011; 30: 292–305. 10.1016/j.jaa.2011.06.002

[33] Ramirez IO, Galili E, Be’eri R, Golan D, Krakovsky M, Dayan A, et al. Heated mud bricks in submerged and coastal Southern Levant Pre-Pottery Neolithic C and Late Pottery Neolithic/Early Chalcolithic settlements: Diachronic changes in technology and their social implications. J Archaeol Sci Rep. 2020; 30: 102220. 10.1016/j.jasrep.2020.102220

[34] Harvey EL, Fuller DQ. Investigating crop processing using phytolith analysis: The example of rice and millets. J Archaeol Sci. 2005; 32: 739–752. 10.1016/j.jas.2004.12.010

[35] Grave P, Kealhoffer L. Assessing bioturbation in archaeological sediments using soil morphology and phytolith analysis. J Archaeol Sci. 1999; 26: 1239–1248. 10.1006/jasc.1998.0363

[36] Madella M, Lancelotti C. Taphonomy and phytoliths: A user manual. Quat Int. 2012; 275: 76–83. 10.1016/j.quaint.2011.09.008

[37] Vrydaghs L, Ball T, Devos Y. Beyond redundancy and multiplicity. Integrating phytolith analysis and micromorphology to the study of Brussels Dark Earth. J Archaeol Sci. 2016; 68: 79–88. 10.1016/j.jas.2015.09.004

[38] Vrydaghs L, Devos Y. Phytolith Analysis on Soil and Ceramic Thin Sections. In: Smith C, editor. Encyclopedia of Global Archaeology. Cham: Springer; 2018. Living Edition. 10.1007/978-3-319-51726-1_3286-1

[39] Petö Á, Vrydaghs L. Phytolith analysis of ceramic thin-sections. First taphonomical insights from experiments with vegetal tempering. In: Sibbesson E, Jervis B, Coxon S, editors. Insight from innovation. New light on Archaeological ceramics. Papers presented in honor of Professor David Peacock’s, contributions to archaeological ceramic studies. Southampton: The Highfield Press; 2016. p. 57–73.

[40] Devos Y, Vrydaghs L. Looking at Phytoliths in Archaeological Soil and Sediment Thin Sections. Environ. Archaeol. 2023; p. 1–16. 10.1080/14614103.2023.2234155

[41] Vrydaghs L, De Paepe P, Rutten K, Haerinck E. A space of exchanges. Phytolith analysis of ceramic thin sections from ed-Dur (Umm al-Qaiwain, U.A.E.). In: Madella M, Lancelotti C, Savard M, editors. Ancient plants and People. Contemporary trends in archaeobotany. Tucson: Arizona University Press; 2014. p. 26–46.

[42] Strabo. Geography. Book 14, chapter 6, section 3.2.

[43] Plutarch. Lucullus, chapter 31.

[44] Lichtenberger A, Schreiber T, Zardaryan MH. Artaxata in Armenien. Eine hellenistische Metropole in der Ararat Ebene. Antike Welt. 2022; 22(5): 41–47.

[45] Ting C, Erhardt S, Gyulamiryan HA, Lichtenberger A, Muradyan SR, Schreiber T, et al. The Artaxiad capital of ceramic: Exploring the changing local pottery production and exchange at Artaxata (Armenia) from the 2nd century BCE to 1st century CE. Archaeol Res Asia. 2023; 34: 100444. 10.1016/j.ara.2023.100444

[46] Zardaryan MH. The Early Iron Age settlement of Artashat and problems of chrono-topography of the site (pre-Classical period). Aramazd. 2018; 12(2): 105–145.

[47] Lichtenberger A, Meyer C, Zardaryan MH. Report on the 2018 Magnetic Prospection at Artaxata/Artashat in Armenia. Archäologischer Anzeiger. 2019; 2: 70–89. 10.52971/18294316

[48] Lichtenberger A, Schreiber T, Zardaryan MH. The wall decoration of a plastered building in Artaxata-Artashat in the Ararat plain of Armenia. Parthica. 2021; 23: 79–96.

[49] Gyulamiryan HA, Muradyan SR, Zardaryan MH, Lichtenberger A, Schreiber T. The Armenian-German Archaeological Project: Results from the Excavations in Artaxata 2018–2021, ԳիտականԱշխատություններ. 2021; 2(24): 5–18. 10.52971/18294316

[50] Lichtenberger A, Zardaryan MH. Preliminary Report of the 2018 campaign of the Armenian-German Artaxata Project. Boreas. 2018/19; 41–42: 39–48.

[51] Tonikian A. The Layout of Artashat and Its Historical Development. Mesopot. 1992; 27: 161–187.

[52] Whitbread IK. A proposal for the systematic description of thin sections towards the study of ancient technology. In: Maniatis Y, editor. Archaeometry Proceedings of the 25th International Symposium (Held in Athens from 19 to 23 May 1986). Amsterdam: Elsevier; 1989. p. 127–138.

[53] Whitbread IK. Greek transport amphorae: a petrological and archaeological study. Athens: British School at Athens; 1995.

[54] Quinn PS. Ceramic petrography: The interpretation of archaeological pottery related artefacts in thin section. Oxford: Archaeopress; 2013.

[55] Bullock P, Fedoroff N, Jongerius A, Stoops G, Tursina T. Handbook for soil thin section description. Wolverhampton: Waine Research; 1985.

[56] Nicosia C, Stoops G. Archaeological soil and sediment micromorphology. Hoboken: John Wiley & Sons; 2017.

[57] Carpentier F, Vandermeulen B. High-Resolution Photography for Soil Micromorphology Slide Documentation. Geoarchaeology. 2016; 31(6): 603–607. 10.1002/gea.21563

[58] Stoops G. Guidelines for Analysis and Description of Soil and Regolith Thin Sections. Madison, Wisconsin: Soil Science Society of America; 2003.

[59] Vrydaghs L, Devos Y. Phytolith analysis of soil and ceramic thin sections. In: Smith C, editor. Encyclopedia of Global Archaeology. Amsterdam: Elsevier; 2018. p. 1–7. 10.1007/978-3-319-51726-1_3286-1

[60] ICPT (Neumann K, Strömberg CAE, Ball TB, Albert RM, Vrydaghs L, Scott Cummings L). International Code for Phytolith Nomenclature (ICPN) 2.0. Ann Bot. 2019a; 124(2): 189–199. doi: 10.1093/aob/mcz064 31334810 PMC6758648

[61] ICPT (Neumann K, Strömberg CAE, Ball TB, Albert RM, Vrydaghs L, Scott Cummings L). International Code for Phytolith Nomenclature (ICPN) 2.0. Ann Bot. 2019b; 124(2): 189–199. doi: 10.1093/aob/mcz064 31334810 PMC6758648

[62] Kaczorek D, Vrydaghs L, Devos Y, Petö A, Effland WR. Biogenic Siliceous Features. In: Stoops G, Marcelino V, Mees F, editors. Interpretation of micromorphological features of soils and regoliths. Amsterdam, Oxford, Cambridge: Elsevier; 2010. p. 157–176.

[63] Karkanas P. Microscopic deformation structures in archaeological contexts. Geoarchaeology. 2019; 34: 15–29. 10.1002/gea.21709

[64] Karkanas P. Identification of lime plaster in prehistory using petrographic methods: A review and reconsideration of the data on the basis of experimental and case studies. Geoarchaeology. 2007; 22(7): 775–796. 10.1002/gea.20186

[65] Macphail R, Goldberg P. Applied Soils and Micromorphology in Archaeology. Cambridge: Cambridge University Press; 2017. 10.1017/9780511895562

[66] Lambert RSJ, Holland JG, Owen PF. Chemical Petrology of a suite of calc-alkaline lavas from Mt. Ararat, Turkey. J Geol. 1974; 82: 419–438.

[67] Yilmaz Y, Güner Y, Saroğlu F. Geology of the quaternary volcanic centres of the east Anatolia. J Volcanol Geotherm Res. 1998; 85: 173–210.

[68] Devolder M. Construire en Crète minoenne: Une approche énergétique de l’architecture néopalatiale. Leuven: Peeters; 2013.

[69] Rosen M. Cities of Clay: the geoarchaeology of Tells. Chicago: University of Chicago Press; 1986.

[70] Shao M, Li L, Sijing W, Enzhi W, Zuixiong L. Deterioration mechanisms of building materials of Jiaohe ruins in China. J Cult Her. 2013; 14(1): 38–44. 10.1016/j.culher.2012.03.006

[71] Zaremba M, Trzciński J, Szczepański T, Bobrowska A, Welc F. Influence of Deterioration on the Preservation of Mud Brick Architecture Based on the Monuments from the Tell El-Retaba Archaeological Site. Int J Conserv Sci. 2021; 12(1): 67–86.

[72] Sapir Y, Avraham A, Faust A. Mud-brick composition, archeological phasing and pre-planning in Iron Age structures: Tel ‘Eton (Israel) as a test-case. Archaeol Anthropol Sci. 2018; 10: 337–350. 10.1007/s12520-016-0350-z

