# Supplementary material for: Exploring mudbrick architecture and its re-use in Artaxata, Armenia, during the 1st millennium BC. A multidisciplinary study of earthen architecture in the Armenian Highlands
Source: PLoS One. 2023 Oct 13;18(10):e0292361. doi: 10.1371/journal.pone.0292361 (PMC10575515; doi:10.1371/journal.pone.0292361)
Supplement: S1 File — Supplementary information in relation to 14C dating. (DOCX) [file pone.0292361.s001.docx]

14C dating parameters

1. Charcoal, Wood, Sediment, Peat, Seed, Food, Plants: The samples are pre-treated using the ABA-Method (Acid/Base/Acid, HCl/NaOH/HCl). The insoluble fraction is used for further treatment
2. ^14^C is analyzed using a MICADAS-type AMS system in-house. The isotopic ratios ^14^C/^12^C and ^13^C/^12^C of samples, calibration standard (Oxalic Acid-II), blanks and control standards are measured simultaneously in the AMS.
3. ^14^C-ages are normalized to δ^13^C=-25‰ (Stuiver & Polach, 1977) and calibrated using the dataset IntCal20 and software Oxcal.
4. Calibration graphs are generated using the software OxCal.
