# Supplementary material for: Exploring mudbrick architecture and its re-use in Artaxata, Armenia, during the 1st millennium BC. A multidisciplinary study of earthen architecture in the Armenian Highlands
Source: PLoS One. 2023 Oct 13;18(10):e0292361. doi: 10.1371/journal.pone.0292361 (PMC10575515; doi:10.1371/journal.pone.0292361)
Supplement: S3 File — Summary of the petrographic fabrics identified in Artaxata. (DOCX) [file pone.0292361.s003.docx]

| Sample number | PF | *c:f:v* | Particle size | sorting | color | Minerals and rock fragments | Voids | Organic inclusions |
| --- | --- | --- | --- | --- | --- | --- | --- | --- |
| AA1 | 1.2 | 20:75:5 | medium silt to very coarse sand | Poorly sorted | Yellowish brown in XP and light brown in PPL | Sub-angular to sub-rounded igneous rocks (basalt, andesite), monocrystalline quartz, calcite, plagioclase feldspar, pyroxenes, and iron-oxide nodules. Very rare microfossils (ostracods) | Meso channels and vesicles | Remains of vegetal temper |
| AA2 | 1.2 | 20:75:5 | medium silt to very coarse sand | Poorly sorted | Yellowish brown in XP and light brown in PPL | Sub-angular to sub-rounded igneous rocks (basalt, andesite), monocrystalline quartz, serpentine, calcite, plagioclase feldspar, pyroxenes, and iron-oxide nodules. Very rare microfossils (foraminifera) and shells. | Meso channels, vughs, and vesicles | Remains of vegetal temper // Charcoal |
| AA3 | 1.1 | 40:55:5 | medium silt to very coarse sand | Poorly sorted | Yellowish brown in XP and light brown in PPL | Angular to sub-rounded igneous rocks (basalt, andesite, volcanic glass), mono and polycrystalline quartz, plagioclase feldspar, calcite, pyroxenes, grey clay pellets, and iron-oxide nodules. Very rare microfossils (gastropods). | Micro planar voids and vesicles | Remains of vegetal temper |
| AA4 | 1.1 | 40:55:5 | medium silt to very coarse sand | Poorly sorted | Yellowish brown in XP and light brown in PPL | Angular to sub-rounded igneous rocks (basalt, volcanic glass), mono and polycrystalline quartz, calcite, plagioclase feldspar, pyroxenes, grey and brown clay pellets (including lumps), and iron-oxide nodules. | Micro vesicles | Remains of vegetal temper // Charcoal |
| AA5 | 1.1 | 40:55:5 | medium silt to very coarse sand | Poorly sorted | Yellowish brown in XP and light brown in PPL | Angular to sub-rounded igneous rocks (basalt, volcanic glass), mica schist, mono and polycrystalline quartz, calcite, plagioclase feldspar, pyroxenes, grey and brown clay pellets, and iron-oxide nodules. | Meso channels and vesicles | - |
| AA6 | 1.1 | 40:55:5 | medium silt to granules | Poorly sorted | Yellowish brown in XP and light brown in PPL | Sub-rounded igneous rocks (basalt, volcanic glass), mono and polycrystalline quartz, limestone, serpentine, plagioclase feldspar, pyroxenes, brown clay pellets (including lumps), and iron-oxide nodules. Very rare microfossils (ostracods) | Meso vughs and vesicles | - |
| AA7 | 1.1 | 40:53:7/8 | medium silt to granules | Poorly sorted | Yellowish brown in XP and light brown in PPL | Sub-angular to sub-rounded igneous rocks (basalt, andesite, volcanic glass), metamorphic rocks, monocrystalline quartz, plagioclase feldspar, calcite, pyroxenes, brown clay pellets, and iron-oxide nodules. | Meso channels, vughs, and vesicles | Charcoal |
| AA8 | 1.1 | 40:55:5 | medium silt to very coarse sand | Poorly sorted | Yellowish brown in XP and light brown in PPL | Angular to sub-rounded igneous rocks (basalt, pumice), monocrystalline quartz, metamorphic rocks, zoned plagioclase feldspars, pyroxenes, brown clay pellets (including lumps), and iron-oxide nodules. | Meso planar voids, micro channels and vesicles | Remains of vegetal temper |
| AA9 | 1.1 | 40:55:5 | medium silt to granules | Poorly sorted | Yellowish brown in XP and light brown in PPL | Sub-angular to rounded igneous rocks (basalt, pumice), metamorphic rocks (granules of mica schist), quartz, plagioclase feldspars, calcite, pyroxenes, brown clay pellets, and iron-oxide nodules. | Meso planar voids and vughs, micro vesicles | - |
| AA10 | 1.1 | 40:55:5 | medium silt to granules | Poorly sorted | Yellowish brown in XP and light brown in PPL | Sub-rounded igneous rocks (volcanic glass, andesite, basalt) and brown clay pellets (including lumps). Other inclusions are quartz, plagioclase feldspars, calcite, metamorphic rocks, serpentine, and iron-oxide nodules. Very rare microfossils (Globigerina). | Meso and micro channels and voids | Charcoal |
| AA11 | 1.2 | 20:75:5 | medium silt to granules | Poorly sorted | Yellowish brown in XP and light brown in PPL | Angular to sub-rounded igneous (basalt) and metamorphic rocks (mica schist and phyllite), quartz, plagioclase feldspars, calcite, pyroxenes, brown clay pellets, and iron-oxide nodules. | Meso channels, micro vughs and voids | - |
| AA12 | 1.2 | 20:75:5 | medium silt to very coarse sand | Poorly sorted | Yellowish brown in XP and light brown in PPL | Sub-rounded to sub-angular igneous rocks (basalt, volcanic glass), monocrystalline quartz, metamorphic rocks, plagioclase feldspars, calcite, pyroxenes, brown clay pellets, iron-oxide nodule and very rare shells. | Meso planar voids, channels and vesicles | - |
| AA13 | 1.2 | 20:75:5 | medium silt to granules | Poorly sorted | Yellowish brown in XP and light brown in PPL | Angular to sub-rounded igneous rocks (basalt, andesite), serpentine, lime nodules, mono and polycrystalline quartz, calcite, plagioclase feldspars, pyroxenes, brown clay pellets (including lumps), iron-oxide nodule, and chert. | Meso planar voids, channels and vesicles | - |
| AA14 | 1.1 | 40:55:5 | medium silt to very coarse sand | Poorly sorted | Yellowish brown in XP and light brown in PPL | Sub-angular to sub-rounded igneous rocks (basalt, pumice, volcanic glass), quartz, calcite, plagioclase feldspars, serpentine, brown clay pellets, iron-oxide nodules, and very rare microfossils (Echinoid and Globigerinoides) | Micro planar voids and channels. | - |
| AA15 | 1.3 | 40:53:7/8 | medium silt to granules | Poorly sorted | Yellowish brown in XP and light brown in PPL | Frequent presence of metamorphic rocks (mainly mica schists but also phyllite) + igneous rocks.  Less freq.: calcite quartz, plagioclase feldspar, pyroxenes, and iron-oxide nodules. Very rare microfossils (Planktic Foraminifera, Globigerina). | Meso/micro vughs and channels | - |
| AA16 | 1.1 | 40:55:5 | medium silt to very coarse sand | Poorly sorted | Yellowish brown in XP and light brown in PPL | Sub-angular to rounded igneous rocks (basalt, andesite, pumice), metamorphic rocks (phyllite), quartz, calcite, plagioclase feldspar, pyroxenes, and iron-oxide nodules. | Meso/micro vesicles and vughs | - |
| AA17 | 1.2 | 20:75:5 | medium silt to granules | Poorly sorted | Yellowish brown in XP and light brown in PPL | Sub-rounded to rounded igneous rocks, mono and polycrystalline quartz, calcite, plagioclase feldspar, pyroxenes, brown clay pellets, biotite, and iron-oxide nodules. Very rare microfossils (Foraminifera, Gastropod) | Meso/micro vesicles and vughs | Remains of vegetal temper // Charcoal |
| AA18 | 1.1 | 40:55:5 | medium silt to granules | Poorly sorted | Yellowish brown in XP and light brown in PPL | Sub-rounded to Sub-angular igneous rocks, metamorphic rocks, brown clay pellets (including lumps), mono and polycrystalline quartz, calcite, feldspar, biotite, pyroxenes, and iron-oxide nodules. | Meso and micro vesicles | Charcoal |
| AA19 | 1.2 | 20:75:5 | medium silt to very coarse sand | Poorly sorted | Yellowish brown in XP and light brown in PPL | Sub-rounded to angular igneous rocks (basalt, pumice), metamorphic rocks, mono and polycrystalline quartz, calcite, plagioclase feldspar, brown clay pellets, pyroxenes, and iron-oxide nodules. | Meso channels and micro vesicles | - |
| AA20 | 1.2 | 20:75:5 | medium silt to very coarse sand | Poorly sorted | Yellowish brown in XP and light brown in PPL | Sub-rounded to Sub-angular igneous rocks, quartz, calcite, brown clay pellets, limestone, metamorphic rocks, pyroxenes, iron-oxide nodules, and shells. | Meso vughs and micro channels and vesicles | Remains of vegetal temper // Charcoal |
| AA21 | 1.1 | 40:55:5 | medium silt to granules | Poorly sorted | Yellowish brown in XP and light brown in PPL | Sub-rounded to Sub-angular igneous rocks (basalt, volcanic glass, andesite), limestone, quartz, calcite, plagioclase feldspars, serpentine, brown (including lumps) and grey clay pellets, iron-oxide nodules, biotite, pyroxenes, and very rare microfossils (Benthic foraminifera) | Micro vesicles and channels | - |
| AA22 | 1.2 | 20:75:5 | medium silt to granules | Poorly sorted | Yellowish brown in XP and light brown in PPL | Sub-rounded to angular igneous rocks (basalt, volcanic glass, pumice), phyllite, quartz, calcite, plagioclase feldspars, iron-oxide nodules, serpentine, pyroxenes, and brown clay pellets. | Micro vesicles and channels | - |
| AA24 | 1.2 | 20:75:5 | medium silt to very coarse sand | Poorly sorted | Yellowish brown in XP and light brown in PPL | Angular to sub-rounded igneous rocks (basalt, pumice), metamorphic rocks, mono and polycrystalline quartz, calcite, iron-oxide nodules, pyroxenes, and brown clay pellets. Very rare microfossils (Echinoid, planktic foraminifera) and shells. | Meso vughs, micro vesicles and channels | - |
| AA25 | 1.2 | 20:75:5 | medium silt to very coarse sand | Poorly sorted | Yellowish brown in XP and light brown in PPL | Angular to sub-rounded igneous rocks (basalt, andesite), metamorphic rocks, limestone, mono and polycrystalline quartz, calcite, iron-oxide nodules, serpentine, and brown clay pellets (including lumps). Very rare microfossils (Globigerina). | Meso channels and micro voids | Remains of vegetal temper |
| AA26 | 1.2 | 20:75:5 | medium silt to very coarse sand | Poorly sorted | Yellowish brown in XP and light brown in PPL | Sub-rounded to Sub-angular igneous rocks (basalt, volcanic glass), limestone, monocrystalline quartz, calcite, metamorphic rocks, plagioclase feldspars, pyroxenes, brown clay pellets, and iron-oxide nodules. | Meso channels and micro voids | - |
| AA27 | 1.2 | 20:75:5 | medium silt to granules | Poorly sorted | Yellowish brown in XP and light brown in PPL | Angular to sub-rounded igneous rocks (basalt, pumice), calcite, monocrystalline quartz, metamorphic rocks, plagioclase feldspars, serpentine, biotite, brown clay pellets (including lumps), pyroxenes, and iron-oxide nodules | Meso/micro channels and voids | Remains of vegetal temper |
| AA28 | 1.1 | 40:55:5 | medium silt to granules | Poorly sorted | Yellowish brown in XP and light brown in PPL | Angular to sub-rounded igneous rocks (andesite, basalt, volcanic glass), metamorphic rocks (mainly phyllite), brown clay pellets (including lumps), calcite, monocrystalline quartz, plagioclase feldspars, serpentine, pyroxenes, and iron-oxide nodules. Very rare microfossils (Planktic foraminifera, Globigerina). | Micro vughs and voids | - |
| AA30 | 1.1 | 40:53:7/8 | medium silt to granules | Poorly sorted | Yellowish brown in XP and light brown in PPL | Sub-rounded to sub-angular igneous rocks (basalt, pumice, andesite), limestone grains, metamorphic rocks, mono and polycrystalline quartz, brown clay pellets, plagioclase feldspars, serpentine, pyroxenes, and iron-oxide nodules. Very rare microfossils (Planktic foraminifera, Gastropods). | Meso plannar voids, channels, vughs and voids | Remains of vegetal temper |
| AA31 | 2 | 30:60:10 | medium sand to medium silt | Moderately sorted | Orangey brown in XP and intense brown in PPL | Rounded to angular igneous (andesite, basalt, volcanic glass) and metamorphic rocks.  Less freq.: calcite, plagioclase feldspar, quartz, serpentine, biotite, pyroxenes, and iron-oxide nodules. | Planar voids and vughs (veg. temp.) + thin channels | Remains of vegetal temper |
| AA32 | 1.1 | 40:55:5 | medium silt to granules | Poorly sorted | Yellowish brown in XP and light brown in PPL | Sub-rounded to Sub-angular igneous rocks (basalt, pumice), metamorphic rocks (mainly mica schist), limestone, mono and polycrystalline quartz, calcite, zoned plagioclase feldspars, brown clay pellets, pyroxenes, and iron-oxide nodules. Very rare microfossils (Planktic foraminifera, Globigerina). | Micro channels and voids | - |
| AA34 | 1.2 | 20:75:5 | medium silt to granules | Poorly sorted | Yellowish brown in XP and light brown in PPL | Sub-rounded to Sub-angular igneous rocks (basalt, andesite, pumice), metamorphic rocks, brown grey pellets (including lumps), mono and polycrystalline quartz, calcite, plagioclase feldspars, serpentine, pyroxenes, iron-oxide nodules, and chert. Very rare microfossils (Planktic foraminifera, Globigerina). | Meso channels and planar voids, micro vesicles | Remains of vegetal temper // Charcoal |
