# Supplementary material for: Exploring mudbrick architecture and its re-use in Artaxata, Armenia, during the 1st millennium BC. A multidisciplinary study of earthen architecture in the Armenian Highlands
Source: PLoS One. 2023 Oct 13;18(10):e0292361. doi: 10.1371/journal.pone.0292361 (PMC10575515; doi:10.1371/journal.pone.0292361)
Supplement: S4 File — Summary of the micromorphological analysis in Artaxata. (DOCX) [file pone.0292361.s004.docx]

| sample number | particle size | sorting | birefringence fabric | color | micro-structure | porosity | voids | minerals and rock fragments | sediment aggregates | organic inclusions |
| --- | --- | --- | --- | --- | --- | --- | --- | --- | --- | --- |
| AA34 | silt loams | moderately sorted | speckled to crystallitic or undifferentiated | yellowish brown | vughy with channels few laminated aggregates | 20% | subrounded to rounded and polyconcave voids, reaching 1 cm in diameter; locally banded, parallel oriented and distributed elongated channels are also recorded, locally curved, in irregular distribution and orientation or spiral deformation | subrounded to subangular quartz, plagioclase feldspar, igneous rocks, pumice | 1) randomly distributed subrounded dark yellowish-brown aggregates with dense calcitic cementing fabric, shrinkage cracks, few vesicles and irregular voids with smooth walls  2) irregular aggregates of immiscible sediments 3) aggregates of laminated sediments |  |
| AA8 | silt loams | moderately sorted | speckled to crystallitic or undifferentiated | yellowish brown | Vughy with fine channels | 20-30% | subrounded to rounded voids and elongated channels randomly distributed, locally curved, in irregular distribution and orientation | quartz, plagioclase feldspar, igneous rocks, and chert | irregular aggregates of immiscible sediments | spherulites |
