# Supplementary material for: Exploring mudbrick architecture and its re-use in Artaxata, Armenia, during the 1st millennium BC. A multidisciplinary study of earthen architecture in the Armenian Highlands
Source: PLoS One. 2023 Oct 13;18(10):e0292361. doi: 10.1371/journal.pone.0292361 (PMC10575515; doi:10.1371/journal.pone.0292361)
Supplement: S5 File — Supplementary information from phytoliths analysis: Table 1. Illustrations of Distribution Patterns (1.1 Isolated and 1.2 Clustered); Table 2. Illustration of phytoliths observed in the clay fabric. (DOCX) [file pone.0292361.s005.docx]

**Table 1. Illustrations of Distribution Patterns (Isolated and Clustered)**

1.1 Isolated phytoliths

| 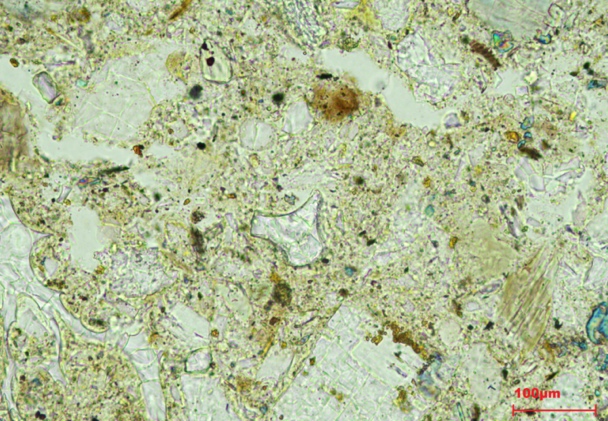 |  | 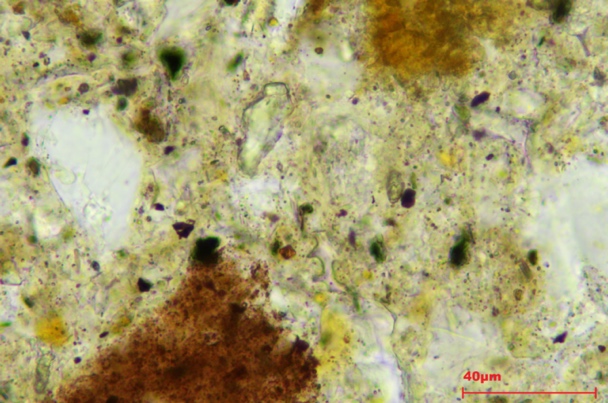 |
| --- | --- | --- |
| Bulliform flabellate, AA 7; x500 PPL. |  | Elongate dentate, AA 7; x500 PPL. |
| 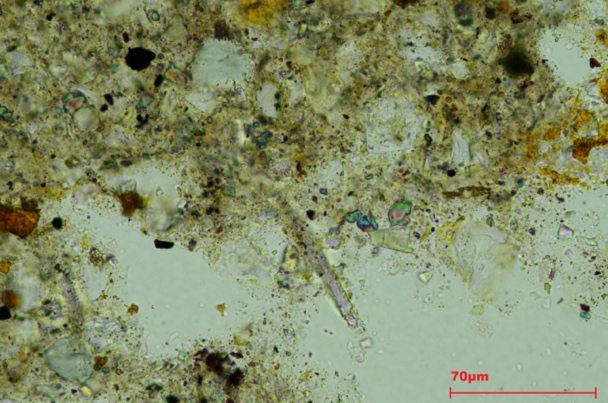 |  | 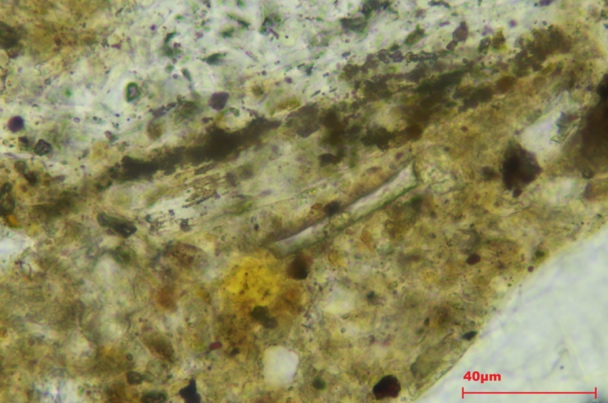 |
| Elongate entire_2, AA 124; x500 PPL. |  | Trapezoid, AA 117; x500 PPL. |

1.2 Clustered phytoliths

| 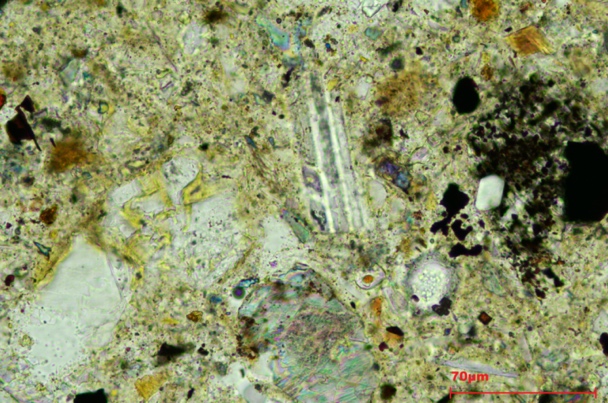 |  | 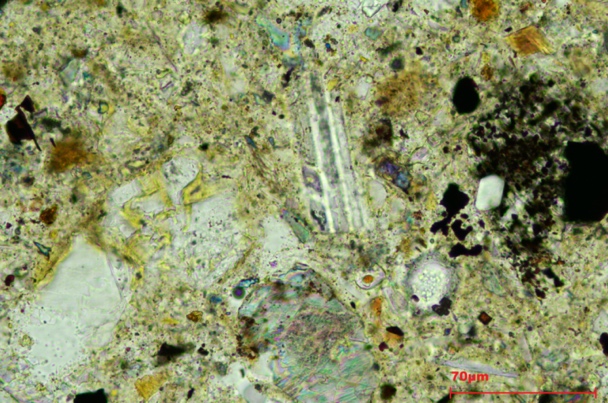 |
| --- | --- | --- |
| Focus 1 on a Cluster made of Articulated phytoliths (blue arrow) and Unidentified phytolith (red arrow); AA 7; x500 PPL |  | Focus 2 on a Cluster made of Articulated phytoliths and Unidentified phytolith, putting into evidence a second Unidentified phytolith (green arrow); AA 7; x500 PPL |
| 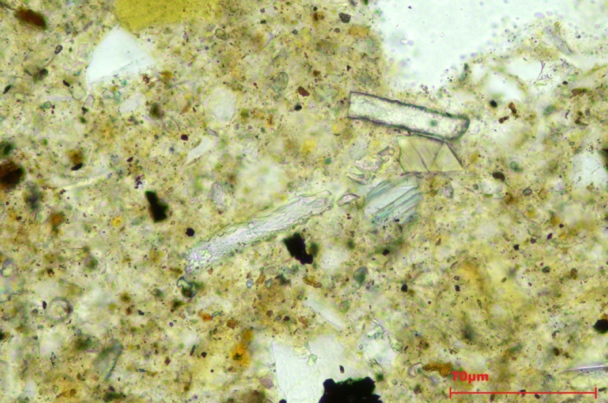 |  | 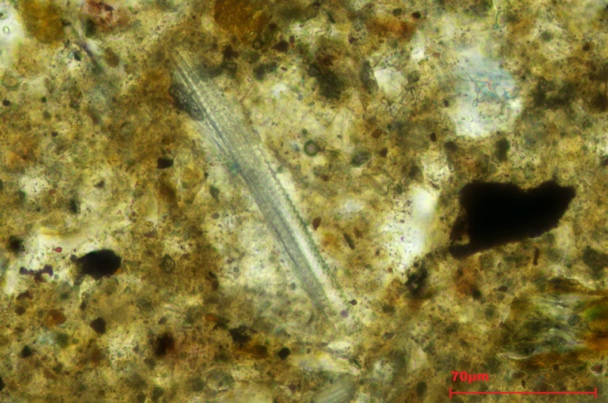 |
| Clustered Elongate entire. AA 17; x500 PPL |  | Cluster made of a Tracheary and a Rondel (red arrow). AA 117; x500 PPL |

**Table 2. Illustration of some phytoliths observed in the clay fabric**

| 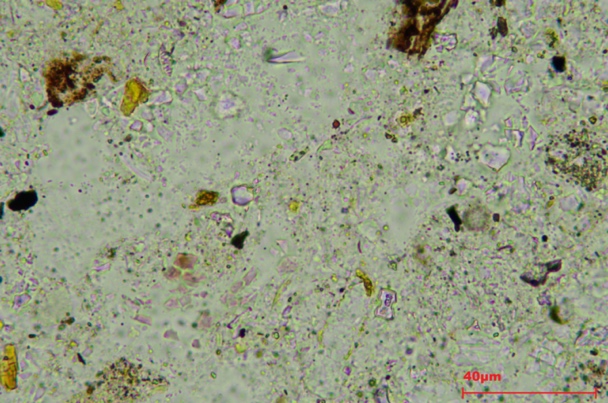 |  | 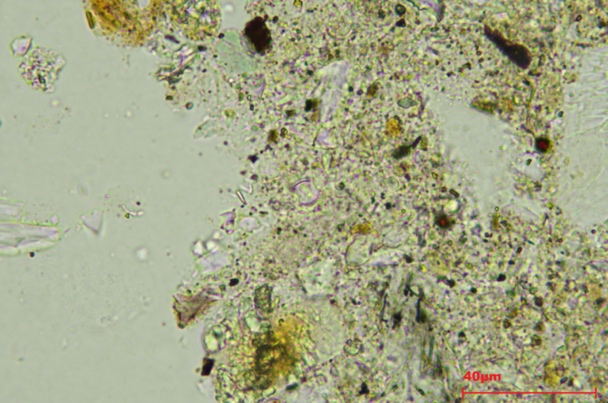 |
| --- | --- | --- |
| Bilobate (red arrow), AA 7; x500 PPL |  | Saddle (red arrow), AA 7; x500 PPL |
| 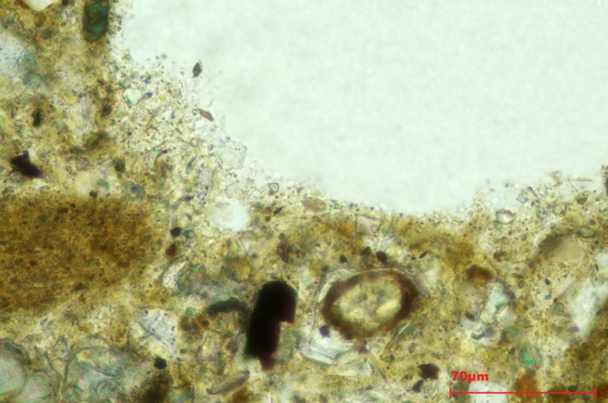 |  | 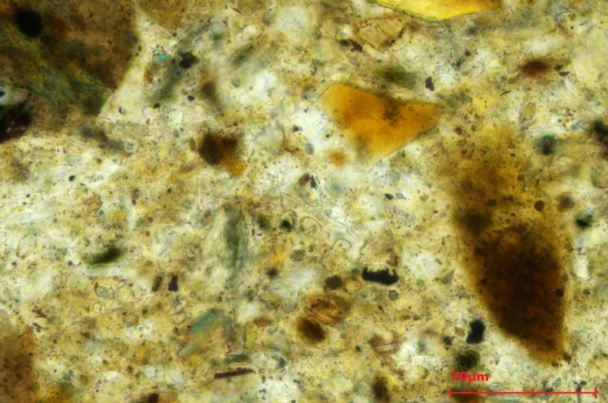 |
| Rondel (red arrow), AA 124; x500 PPL |  | Elongate dentate (red arrow), AA 117; x500 PPL. |
